# Universal quenching of common fluorescent probes by water and alcohols†

**DOI:** 10.1039/d0sc05431c

**Published:** 2020-11-19

**Authors:** Jimmy Maillard, Kathrin Klehs, Christopher Rumble, Eric Vauthey, Mike Heilemann, Alexandre Fürstenberg

**Affiliations:** Department of Physical Chemistry, University of Geneva 1211 Geneva Switzerland alexandre.fuerstenberg@unige.ch; Department of Inorganic and Analytical Chemistry, University of Geneva 1211 Geneva Switzerland; Institute for Physical and Theoretical Chemistry, Goethe University Frankfurt 60438 Frankfurt am Main Germany heilemann@chemie.uni-frankfurt.de

## Abstract

Although biological imaging is mostly performed in aqueous media, it is hardly ever considered that water acts as a classic fluorescence quencher for organic fluorophores. By investigating the fluorescence properties of 42 common organic fluorophores recommended for biological labelling, we demonstrate that H_2_O reduces their fluorescence quantum yield and lifetime by up to threefold and uncover the underlying fluorescence quenching mechanism. We show that the quenching efficiency is significantly larger for red-emitting probes and follows an energy gap law. The fluorescence quenching finds its origin in high-energy vibrations of the solvent (OH groups), as methanol and other linear alcohols are also found to quench the emission, whereas it is restored in deuterated solvents. Our observations are consistent with a mechanism by which the electronic excitation of the fluorophore is resonantly transferred to overtones and combination transitions of high-frequency vibrational stretching modes of the solvent through space and not through hydrogen bonds. Insight into this solvent-assisted quenching mechanism opens the door to the rational design of brighter fluorescent probes by offering a justification for protecting organic fluorophores from the solvent *via* encapsulation.

## Abstract 2

Overtones and combinations of O–H vibrations in the solvent efficiently quench red-emitting fluorophores by resonant energy transfer.

## Graphical abstract

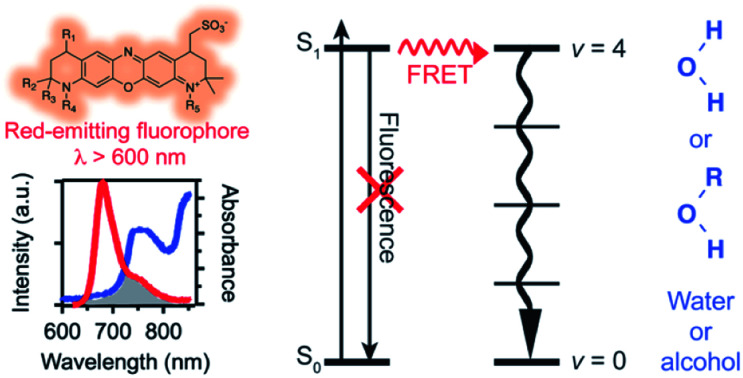

## Introduction

Fluorescence spectroscopy and imaging have become common tools for non-invasive, real-time, and high-resolution visualization and detection of biomolecules and biomolecular processes in cells. They usually rely on the use of extrinsic fluorophores that can be selectively chemically or genetically attached to target biomolecules. In many applications, the ideal fluorophore should not only be bright, *i.e.* display a high molar absorption coefficient and a high fluorescence quantum yield, but also photostable and soluble in water^1^ since most biomolecular processes take place in an aqueous environment. In part owing to a push in the development of organic dyes alongside the advent of single-molecule and super-resolution imaging, a fairly large number of commercially available small organic fluorophores, typically deriving from just a handful of different core structures (*e.g.* cyanines, rhodamines, oxazines), nowadays meet these requirements.^2^

**Fig. 1:**
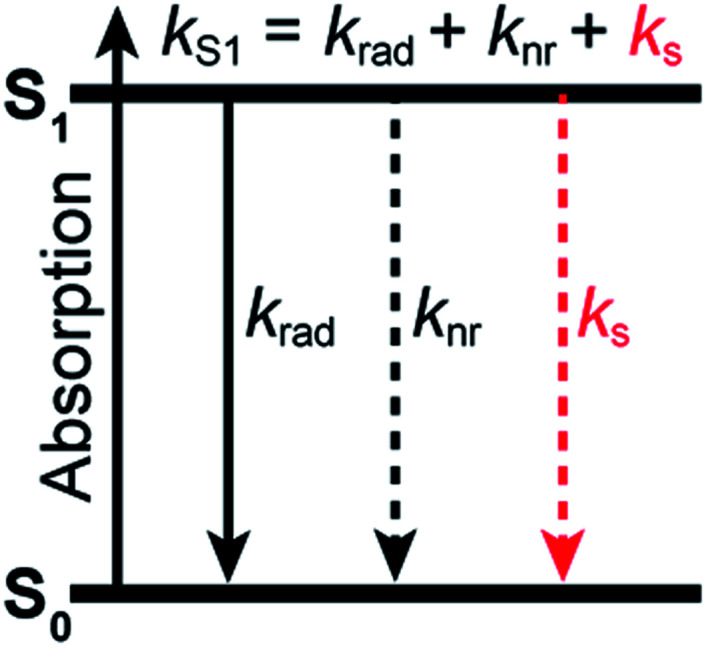
Jablonski diagram summarizing the excited-state dynamics of organic fluorophores in a solvent acting as a quencher. Deactivation of the excited state of the fluorophore (described by the rate constant *k*_S1_) occurs through radiative processes (radiative rate constant *k*_rad_), intramolecular non-radiative processes (non-radiative rate constant *k*_nr_), and quenching interactions with the solvent (solvent-assisted quenching rate constant *k*_s_).

When it comes to the choice of a fluorophore to tag a biomolecule, it is nonetheless often overlooked that water, which is ubiquitous in biological environments, is a weak, yet well-known fluorescence quencher.^3^ Although the specified fluorescence quantum yield of commercial organic dyes is typically in the 0.3–0.9 range, it is not unity and usually lower in water than in organic solvents. Stryer reported over 50 years ago that H_2_O quenches the fluorescence of several organic chromophores containing proton donor groups.^4^ More importantly, he further noted that their fluorescence quantum yield increases significantly in D_2_O, indicating that D_2_O is a much poorer quencher than H_2_O. A few years earlier, it had already been observed that the luminescence of rare earth metal ions was also inhibited in complexes in which H_2_O was present as a ligand in the first coordination sphere, while D_2_O had a much weaker impact.^5,6^ Many subsequent studies have confirmed an isotope effect on the emission quantum yield and excited-state lifetime of chromophores in water^7–18^ and in other solvents.^19–24^ The general picture which emerges is that the solvent, and water in particular, can act as a classic quencher able to compete with radiative and other non-radiative deactivation processes (Fig. 1), and that the solvent-assisted quenching can be selectively and significantly reduced in deuterated solvents. We recently made use of this effect to improve fluorescence imaging of biological samples in a solution environment in which H_2_O has been replaced by D_2_O, demonstrating that this simple substitution leads to a better localization precision in single-molecule based super-resolution imaging.^25,26^

Whereas the mechanism leading to fluorescence quenching by water and to its kinetic isotope effect has been elucidated in detail for inorganic ions such as Eu^3+^ or Tb^3+^ in coordination complexes in which H_2_O or D_2_O are bound as covalent ligands to the luminescent centre,^27–30^ its exact nature remains elusive in the case of organic fluorophores surrounded by loose water as the solvent. For metallic ions, non-radiative energy transfer to stretching vibrations of coordinated or nearby O–H oscillators for which Franck–Condon overlap with energy levels exist is responsible for the quenching, and its efficiency depends on the energy gap between the emissive and ground states of the metal.^31^ With organic molecules, different mechanisms have been called upon depending on the molecular system.^32^ Stryer and others have suggested that excited-state proton transfer between the chromophore and water be responsible for the lower fluorescence quantum yield observed in H_2_O compared with D_2_O.^4,33,34^ Reversible electron transfer between the probe and water has also been discussed as a possible cause for fluorescence quenching by H_2_O^32,35^ but fails to explain the fluorescence enhancement observed in D_2_O. In most instances, hydrogen bonding interactions in the absence of proton transfer or proton-coupled electron transfer are invoked in the so-called hydrogen-bond-assisted non-radiative deactivation of fluorescent dyes in water and in other hydrogen-bonding solvents.^8,36–41^ Recent insight into this process provided by ultrafast transient IR spectroscopy in organic solvents suggests that its efficiency depends on the strength of the H-bonds between the probe and the solvent, with superprotic solvents leading to an efficient, ultrafast quenching.^40^ Therefore, this mechanism cannot either explain why common organic fluorophores such as oxazines or cyanines display a fluorescence enhancement of up to 150% in D_2_O, as recently observed.^25,26^ Ermolaev and Sveshnikova proposed that resonant energy transfer to high-energy vibrations of the solvent be responsible for the quenching of metal ions and small molecules such as NO^2−^ by water, provided a quantum mechanical framework, and hypothesized it could also apply to complex organic molecules, but no general experimental demonstration was provided.^42–44^

We therefore set out to more systematically explore the quenching induced by water and other solvents with 42 organic fluorophores, all of which but one are commercially available and recommended for biological labelling. We observe that the quenching efficiency is stronger for red-emitting than for blue-emitting fluorophores and that it exponentially depends on the S_0_–S_1_ energy gap of the dye. The quenching requires the presence of high-frequency vibrational modes in the solvent *via* OH groups which lead to a weak solvent absorption band around 750 nm and above, but does not depend on the hydrogen-bonding strength of the solvent. Our findings are consistent with a dipole–dipole resonance energy transfer mechanism between the electronically excited dyes and vibrational combination bands of the solvent.

## Results

### Generality of the fluorescence quenching by water and methanol

**Fig. 2:**
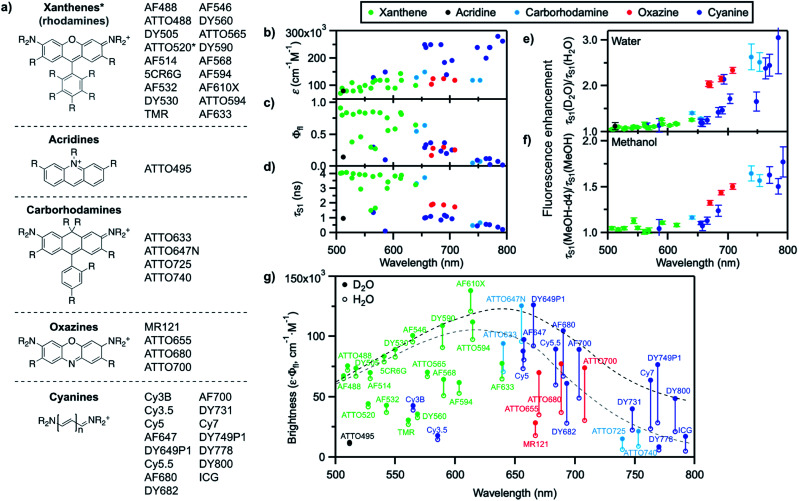
(a) General molecular structures of the investigated dye families and commercial names of the tested fluorophores. Full dye structures are given in Fig. S1–S2.† (b) Molar decadic absorption coefficient of the investigated fluorophores at their peak absorption wavelength (as provided by the manufacturer), (c) measured fluorescence quantum yield in H_2_O, and (d) measured S_1_ excited-state lifetime in H_2_O as a function of their S_0_–S_1_ energy gap. (e) Fluorescence enhancement of the investigated dyes in D_2_O with respect to H_2_O and (f) in MeOH-d4 with respect to MeOH, measured as the ratio of the excited-state lifetime in the deuterated and in the protonated solvent. Error bars reflect the estimated uncertainty arising from the measurement of the excited-state lifetimes. (g) Brightness (*ε*·*Φ*_fl_) distribution of the investigated dyes in H_2_O (hollow circles) and D_2_O (filled circles). The grey dashed line underlines the general trend for the maximal observed brightness in H_2_O, the black dashed line the trend in D_2_O; both lines merely serve as a guide to the eye. In (b–g), every dye is represented by a full circle at the wavelength corresponding to its S_0_–S_1_ energy gap.

We recently reported that the fluorescence quantum yield of several oxazine and cyanine dyes increases by 10–150% in D_2_O compared to H_2_O.^25,26^ Driven by this observation, we set out to investigate other dye classes to look into the generality of the quenching of organic fluorophores by H_2_O and selected 42 common fluorophores recommended for biological imaging (Fig. 2a, S1–S2, Table S1†). The dyes are all related to the xanthene, acridine, carborhodamine, oxazine, and cyanine dye families, have large molar decadic absorption coefficients (*ε*, Fig. 2b), and absorb and emit across the visible spectrum and into the near infrared (Fig. S3†). They show rather small Stokes shifts (<1100 cm^−1^ in water), are known to display little solvatochromism, and are therefore generally not used as environment-sensitive fluorescence probes, but rather as robust intensity markers for the detection of biomolecules. We measured their fluorescence quantum yield, *Φ*_fl_, and excited-state lifetime, *τ*_S1_, in H_2_O and in D_2_O (Fig. 2c, d, S4–S6, Table S2†), and found that both parameters are between 5 and 205% larger for all dyes in D_2_O (Fig. 2e, Table S2†), thereby establishing the generality of the quenching of these common fluorophores by H_2_O as the solvent. It is striking that the dyes showing the largest fluorescence enhancement in D_2_O, and therefore the strongest quenching in H_2_O, are the ones absorbing and emitting at longer wavelengths (>650 nm), whereas the enhancement is only on the order of 5–15% below 600 nm.

We further compared the fluorescence properties in perdeuterated methanol (MeOH-d4) and in methanol (MeOH) for a selection of these dyes (Fig. 2f). The fluorescence quantum yield and lifetime are higher in MeOH than in H_2_O but very similar in MeOH-d4 and in D_2_O (Table S3, Fig. S7–S8†), leading globally to lower enhancement factors in methanol than in water and pointing to less efficient, but nonetheless existing fluorescence quenching in MeOH. The wavelength dependence of the fluorescence enhancement is analogous in methanol and in water, with red-absorbing dyes showing a fluorescence increase of up to 77% in MeOH-d4 compared to MeOH.

A plot of the brightness (defined as *ε*·*Φ*_fl_) of these standard, well-behaved fluorophores in H_2_O as a function of their S_0_–S_1_ energy gap (measured as the midpoint between the absorption and emission maxima on an energy scale) exhibits a bell shape with a maximum in the green-orange spectral region rather than a steady, linear rise (Fig. 2g). Since the molar absorption coefficient of these dyes generally increases with the chromophoric size and thereby with the absorption and emission wavelengths (Fig. 2b, Table S1†), the decrease in brightness at long wavelengths arises from lower fluorescence quantum yield values in the red part of the visible spectrum (Fig. 2c). Although the fluorescence quantum yield is expected to decrease as the S_0_–S_1_ energy gap becomes smaller because of more efficient internal conversion,^45^ the latter process alone cannot explain the strong wavelength dependence of the observed isotope effect. Switching the solvent from H_2_O to D_2_O effectively enables to increase the brightness of the fluorophores by modulating their fluorescence quantum yield, especially at long emission wavelengths (Fig. 2g). Similar conclusions can be drawn for MeOH-d4 with respect to MeOH.

### Solvent-assisted quenching through solvent OH moieties

**Fig. 3:**
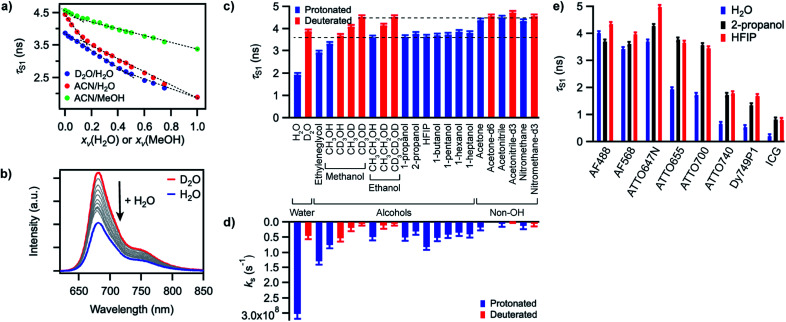
(a) Excited-state lifetime of ATTO655 in D_2_O/H_2_O, ACN/H_2_O, or ACN/MeOH mixtures as a function of the volume fraction *x*_*v*_ of H_2_O or MeOH. Dashed lines serve as guide to the eye to emphasize deviation of the curves from linearity. (b) Evolution of the fluorescence spectrum of ATTO655 in D_2_O upon addition of increasing amounts of H_2_O (at constant dye absorbance). The red curve corresponds to the fluorescence spectrum in pure D_2_O and the blue curve in pure H_2_O. (c) Excited-state lifetime of ATTO655 in various protonated (blue) and partially or fully deuterated (red) solvents. The two dashed lines indicate the lifetime levels observed in alcohols (lower) and in other non-alcoholic polar solvents (upper). (d) Solvent-assisted quenching rate constant *k*_s_ determined for ATTO655 in the same solvents as in (c), with the assumption *k*_nr_ = 0 in acetonitrile-d3. (e) Excited-state lifetime of various fluorophores in H_2_O, 2-propanol, and HFIP. The similarity of the lifetime values in 2-propanol and HFIP indicates that the H-bond strength does not significantly influence the fluorescence quenching process. All error bars reflect uncertainties on the lifetime measurements.

In order to gain better insight into what factors control the quenching efficiency at the molecular level, we investigated the influence of the structure of the solvent molecules on the fluorescence properties of ATTO655, a red-emitting oxazine dye whose fluorescence is efficiently quenched by H_2_O and MeOH.^25^ We determined its fluorescence lifetime in D_2_O and in acetonitrile (ACN) solutions in the presence of increasing quantities of H_2_O or MeOH (Fig. 3a). In D_2_O, the fluorescence lifetime decreases steadily with increasing amounts of H_2_O from 3.9 ns in pure D_2_O to 1.9 ns in pure H_2_O. The fluorescence quantum yield decreases concomitantly (Fig. 3b, S9a†), suggesting a constant radiative lifetime and a purely dynamic quenching process.^46^ In acetonitrile, the change in fluorescence lifetime with varying quantities of H_2_O also closely follows the change in fluorescence quantum yield (Fig. 3a, S9b†). The curves are however biphasic: the decrease of the fluorescence lifetime is steeper at low than at high H_2_O concentrations, pointing to preferential solvation of the electrically charged fluorescent probe by water.^47^ With MeOH as a quencher in ACN, the decrease in excited-state lifetime is also steeper upon addition of small concentrations of MeOH than at high concentrations, indicating that MeOH as well preferentially solvates ATTO655 compared to ACN. The overall decrease of the excited-state lifetime is however smaller than in H_2_O, confirming that MeOH is a less efficient quencher than H_2_O.

A Stern–Volmer analysis of the quenching by H_2_O in D_2_O (Fig. S9a†) yields a dynamic quenching rate constant of 4.8(1) × 10^6^ M^−1^ s^−1^. As the rate constant of diffusion in water is ≈ 6 × 10^9^ M^−1^ s^−1^,^48^ it can be safely concluded that a molecule of H_2_O is a very inefficient quencher *per se*. The quenching process by water is however rendered efficient and observable owing to the high quencher concentration in the direct surroundings of the fluorescent probe (up to 55.5 M).

We next set out to determine which features of the solvent cause it to quench the fluorescence of ATTO655. We measured the fluorescence quantum yield and the excited-state lifetime of ATTO655 in various polar solvents, which included several linear alcohols, non-alcoholic solvents, as well as some partially or fully deuterated solvents (Fig. 3c, S10, Table S4†). The longest excited-state lifetimes (∼4.5 ns) were observed in acetonitrile, nitromethane, and acetone, polar solvents that do not bare any alcohol functional group. Introduction of a single OH group onto a solvent alkyl chain is sufficient to cause partial fluorescence quenching with a reduction of the excited-state lifetime of ATTO655 to ∼3.7–3.8 ns in all linear terminal alcohols from ethanol to 1-heptanol, as well as in 2-propanol. The excited-state lifetime further decreased slightly with smaller methanol, was lowered significantly in the diol ethyleneglycol, and reached its minimum in H_2_O. Altogether, these observations indicate that the density of OH groups around the probe controls the efficiency of the quenching by the solvent molecules. The fact that the dye excited-state lifetime does not depend on the hydrocarbon chain length of the monoalcohols is in line with preferential solvation of the polar probe by OH moieties as observed in MeOH (Fig. 3a).

The importance of OH groups and, in particular, of O–H vibrations in the quenching process was confirmed by measurements of the excited-state lifetime of ATTO655 in partially or fully deuterated solvents (Fig. 3c, S10†). Whereas perdeuteration of the polar non-alcoholic solvents had little or no effect on the fluorescence (lifetime of ∼4.5–4.7 ns), perdeuteration of methanol or ethanol (EtOH) did lead to a significant increase of the excited-state lifetime and of the fluorescence quantum yield back to the level of non-alcoholic solvents. However, although selective deuteration of the hydrogens on the hydrocarbon chain does weakly contribute to restoring the fluorescence (increase of the fluorescence lifetime from 3.3 to 3.7 ns in methanol), selective deuteration of the alcoholic hydrogen leads to a far more significant increase of the excited-state lifetime from 3.3 to 4.1 ns in methanol and from 3.6 to 4.2 ns in ethanol. Interestingly, perdeuteration of water also did not lead to a full restoration of the fluorescence lifetime, suggesting that both CH and OD groups might still be fluorescence quenchers, albeit very weak.

To quantify the efficiency of the quenching by every solvent, we extracted the solvent-assisted quenching rate constant *k*_s_ (Fig. 3d, Table S4†) from the lifetime data by considering the quenching as an intermolecular non-radiative excited-state deactivation process in addition to intramolecular non-radiative processes such as internal conversion (Fig. 1 and see discussion below). We first calculated the radiative rate constant, *k*_rad_, of ATTO655 in every solvent from the fluorescence lifetime and fluorescence quantum yield; as expected, the quantity *k*_rad_/*n*^3^, where *n* is the refractive index, was very similar in all solvents.^49^ With the assumptions that the non-radiative decay constant, *k*_nr_, arising from internal conversion or intersystem crossing is essentially independent of the solvent, and that no solvent-assisted quenching takes place in ACN-d3 (largest fluorescence lifetime), we determined a value of *k*_nr_ ≈ 6.8(8) × 10^7^ s^−1^ for ATTO655. Using the relationship *k*_S1_ = *k*_rad_ + *k*_nr_ + *k*_s_, we were thus able to compute *k*_s_ (Fig. 3d, Table S4†). As expected, the magnitude of *k*_s_ directly mirrors the reduction in excited-state lifetime (Fig. 3c), its value being largest in H_2_O and in ethyleneglycol, and one order of magnitude lower in deuterated and non-alcoholic solvents. The observed kinetic isotope effect is of 6.8 in water and of about 4–5 in MeOH *vs.* MeOD or EtOH *vs.* EtOD. On the other hand, replacement of C–H bonds by C–D bond leads to isotope effects of only 1.4–2.5, clearly indicating the prevalence of the O–H vibration in the quenching process.

### Independence of hydrogen bond strength

Since O–H vibrations seem to be responsible for the quenching of fluorescent probes by the solvent, we further tested whether the strength of the hydrogen bond network around the probe was relevant to the quenching process. Hydrogen-bond-assisted non-radiative deactivation has indeed been observed with other fluorescent probes such as eosin B or acceptor–donor–acceptor systems.^36,40^ Furthermore, D_2_O is believed to form slightly weaker H-bonds than H_2_O.^50^ In order to increase the hydrogen-bond strength between the probe and the solvent, we used hexafluoroisopropanol (HFIP) as one of the very few solvents with a higher hydrogen-bond donating capability (Kamlet–Taft acidity parameter *α* = 1.96) than water (*α* = 1.17).^51,52^ We found the fluorescence quantum yield and the excited-state lifetime of ATTO655 in HFIP (Fig. 3c, Table S4†) to be very similar to the values of these parameters in other alcohols, including in 2-propanol, the non-perfluorinated analogue of HFIP. Independence of the quenching process from the H-bond strength between the probe and the solvent was further confirmed for other fluorophores belonging to the xanthene, oxazine, carborhodamine, and cyanine dye classes for which no reduction in the excited-state lifetime was found in HFIP compared to 2-propanol (Fig. 3e, S11, Table S5†).

## Discussion

### Quenching by the solvent follows an energy gap law

By investigating the fluorescence quantum yield and excited-state lifetime of 42 different dyes belonging to 5 common dye families, we found that they are all more fluorescent in D_2_O than in H_2_O or in MeOH-d4 than in MeOH. Overall, our data indicates that, after excitation of the dyes, and on top of fluorescence emission and standard solvent-independent non-radiative decay processes (essentially internal conversion for these chromophores), an additional solvent-dependent non-radiative relaxation mechanism seems to be operative with higher efficiency for red-emitting than for blue-emitting dyes (Fig. 1). Because of the observed isotope effect upon replacement of OH by OD groups, we must further conclude that the quenching is related to the presence of high-energy vibrational modes in the solvent and that it amounts to an energy transfer from the electronically excited dye (F) to vibrational modes of the solvent (S) as described by the scheme:1
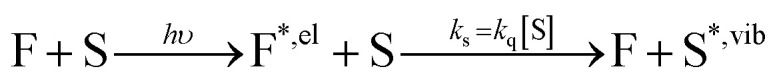


The rate constant associated with the quenching by the solvent, *k*_s_, can be directly related to the bimolecular Stern–Volmer quenching rate constant, *k*_q_, through the quencher concentration (see ESI†).

The total excited-state decay rate constant *k*_S1_ (inverse of the excited-state lifetime *τ*_S1_) of these dyes can be written as the sum of all decay channels, namely *k*_S1_ = *k*_rad_ + *k*_nr_ + *k*_s_, where *k*_rad_ is the radiative rate constant, *k*_nr_ the solvent-independent non-radiative rate constant and *k*_s_ the decay rate constant specifically associated with the quenching by the solvent. Since the solvent-independent non-radiative decay arises mainly from internal conversion with these dyes which do not undergo intersystem crossing efficiently,^53^*k*_nr_ is essentially identical in a protonated solvent (H_2_O or MeOH) and in its deuterated analogue (D_2_O or MeOH-d4).^29^ Also the radiative rate *k*_rad_ does not change between the solvents (Tables S2 and S4†). The difference in the excited-state decay rate constant of any fluorophore in H_2_O and in D_2_O, Δ*k*_S1_, can thus be written as

2 Δ*k*_S1_(water) = *k*_S1_(H_2_O) – *k*_S1_(D_2_O) = *k*_H_2_O_ – *k*_D_2_O_ ≈ *k*_H_2_O_

where *k*_H_2_O_ and *k*_D_2_O_ are the rate constants associated with the quenching process in either isotopic form of the solvent (*k*_s_), and with the assumption that *k*_H_2_O_ ≫ *k*_D_2_O_. Data for the dye ATTO655 in various solvents shows that the latter assumption is experimentally reasonably verified (Fig. 3d, Table S4†). Furthermore, for the dyes for which we performed a Stern–Volmer analysis of their quenching by H_2_O in D_2_O, the relationship between measured *k*_q_ and measured Δ*k*_S1_ ≈ *k*_s_ = *k*_q_[S] holds true within experimental uncertainty. By analogy with water, a similar relationship can be established in methanol:

3 Δ*k*_S1_(methanol) = *k*_S1_(MeOH) − *k*_S1_(MeOH-d4) ≈ *k*_MeOH_

**Fig. 4:**
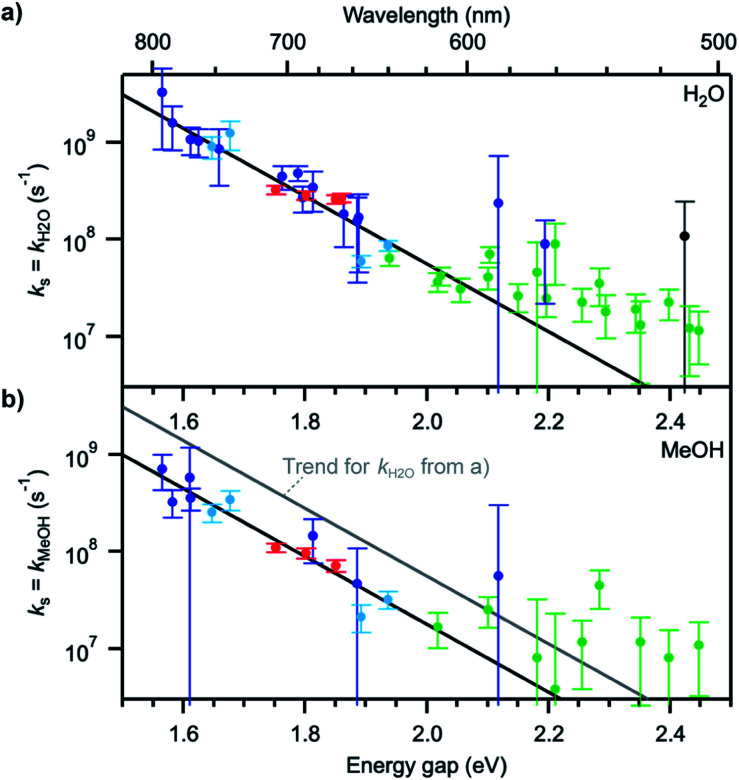
(a) Solvent quenching rate constant in water and (b) in methanol as a function of the S_0_–S_1_ energy gap of each fluorophore. The colour coding of the data points is the same in (a) and (b). Black lines represent trends to the data points at energy gaps up to ∼2.1 eV. The grey line in (b) indicates the trend for *k*_H_2_O_ determined in (a). Error bars were determined from the estimated measurement uncertainty on the fluorescence lifetime.

Plots of Δ*k*_S1_ ≈ *k*_H_2_O_ or Δ*k*_S1_ ≈ *k*_MeOH_ as a function of the S_0_–S_1_ transition energy of the dyes, Δ*E*_00_, the so-called energy gap, show a decaying exponential relationship between the two quantities for small and medium energy gaps, which translates into a straight line on a semi-logarithmic scale (Fig. 4). The non-radiative decay taking selectively place in H_2_O and MeOH thus follows an energy gap law of the type^54^

4 *k*_s_ ≈ Δ*k*_S1_ = *A*exp(−*ζ*Δ*E*_00_)

where *A* is a preexponential term and *ζ* an attenuation factor. For energy gaps up to ∼2.1 eV (∼600 nm), the observed trend can be well reproduced with values of *A* = 5.6 × 10^14^ s^−1^ in H_2_O, *A* = 1.8 × 10^14^ s^−1^ in MeOH, and an attenuation factor *ζ* = 8.1 eV^−1^ in both solvents.

The values for the pre-exponential factor *A* are in line with expectations for a non-radiative process involving vibrations,^54,55^ while the attenuation factor is higher than what was found for the intramolecular non-radiative deactivation of families of aromatic hydrocarbons, azulenes, carotenoids, or fluorenones (*ζ* ≈ 5–7 eV^−1^),^41,55–57^ which typically involves internal conversion or intersystem crossing. The large *ζ* value observed here indicates a stronger dependence of the investigated quenching process on the energy gap and thus predicts an efficient deactivation at small energy gaps, more efficient than internal conversion, and an inefficient deactivation at large energy gaps. The observed deviations from the trend at energy gaps larger than 2.1 eV most probably arise from the fact that other intramolecular deactivation processes, in particular internal conversion, become more efficient than quenching by the solvent and that the measured differences in excited-state lifetime in protonated and deuterated solvents become very small (larger uncertainty on *k*_s_).

### Quenching fluorophores by resonance electronic-to-vibrational energy transfer *via* dipolar coupling

**Fig. 5:**
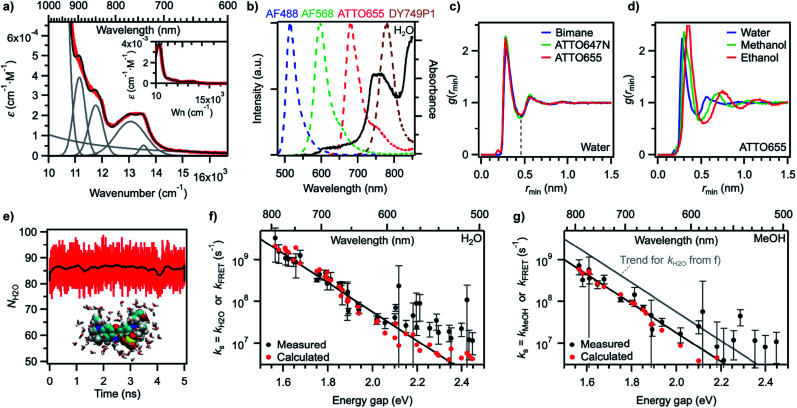
(a) Measured absorption spectrum of H_2_O on the wavenumber scale (red) and reconstructed H_2_O spectrum (black) after decomposition into individual components (grey). Inset: same spectra on a larger *ε* scale (Wn: wavenumber). (b) Overlap between intensity-normalized emission spectra of various fluorophores (dashed lines, left axis) and the absorption spectrum of H_2_O (black line, right axis). (c) Minimum-distance distribution functions for fluorophores of different sizes in water. The dashed line at the first minimum indicates the size of the first solvent shell. (d) Minimum-distance distribution functions for the fluorophore ATTO655 in different solvents. (e) Number of water molecules within the first solvent shell of ATTO655 as a function of simulation time (1 ps time interval). The black trace was obtained by smoothing red data points (250 ps) and highlights that the number is fairly constant. A snapshot of the molecular structure of ATTO655 and the water molecules in the first solvent shell is given as an inset. (f) Measured water quenching rate constant *k*_H2O_ (black circles) and trend line as in Fig. 4a, and calculated dipole–dipole energy transfer rate constant *k*_FRET_ (red circles) in water. (g) Measured methanol quenching rate constant *k*_MeOH_ (black circles) and trend lines as in Fig. 4b, and calculated dipole–dipole energy transfer rate constant *k*_FRET_ (red circles) in methanol.

The energy gap law has been successful, in its weak coupling limit, at correlating the rates of radiationless decay of the lowest excited singlet and triplet states of families of aromatic hydrocarbons with their S_1_–S_0_ and T_1_–T_0_ energy spacings and is very helpful at phenomenologically describing the data. However, by essence, it does not provide any insight into the quenching mechanism, other than that it is compatible with a deactivation of the dye electronic energy mediated by a vibration of the solvent. Vibrations of water are remarkable in that they are the only known vibrations responsible for the natural, visible colour of the molecule to which they belong: several overtones of O–H vibrations in H_2_O absorb in the visible region, with the fourfold combination of fundamental symmetric and/or antisymmetric O–H stretching modes giving rise to an absorption band peaking between 740 and 760 nm and conferring H_2_O its blue-green colour (Fig. 5a, Table S6†).^58,59^ Although weak (*ε*_750_ ≈ 2 × 10^−4^ cm^−1^ M^−1^),^60^ this absorption is detectable over an optical pathlength of 1 cm owing to the high water concentration (55.5 M) leading to maximal absorbance values of about 0.015 for this band.^26^ Also MeOH and other linear alcohols absorb with a similar molar absorption coefficient in the 700–900 nm window (Fig. S12†).^61,62^ Because of the 1.4-fold reduction in the fundamental vibrational frequency when H gets replaced by D, no visible absorption band is detected in D_2_O or in perdeuterated alcohols under the same conditions.^61,63^

The absorption spectrum of H_2_O in the visible-NIR region arises from multiple combination bands of the fundamental vibrational frequencies into which it can be well decomposed using a sum of Gaussian functions centred at or very close to reported combination frequencies (Fig. 5a, Table S6†).^58^ It is striking that the region in which the energy gap law is best followed (600–800 nm in our experiments) corresponds to the spectral region in which H_2_O absorbs, giving rise to a strong overlap of the fluorescence spectra of the best quenched fluorophores with the solvent absorption bands (Fig. 5b). Since the energy transfer process involved in the quenching does not occur through hydrogen bonds (see above) and cannot be accounted for by a Dexter mechanism as no electronic transition takes place in the solvent (the energy acceptor), we wondered whether dipolar coupling between the fluorophore and the solvent could be the mechanism through which energy is transferred (Förster resonance energy transfer, FRET). Resonance vibrational energy transfer by dipolar coupling over distances of up to 4.5 Å (2 water molecules) has been observed before between water and protons^64^ and electronic to vibrational energy transfer between nanocrystals and C–H vibrations in their surrounding matrix environment or dioxygen and water.^65,66,89^ Furthermore, Ermolaev and Sveshnikova correlated the rate of electronic deactivation of the triplet state of organic molecules and the integral of the overlap of the phosphorescence spectrum with the overtone absorption spectrum of water.^42^ In addition, they provided a quantum-mechanical justification for resonant energy transfer between electronic (vibronic) excited states and isoenergetic vibrational oscillators,^43^ obtaining an expression that coincides with the dipole–dipole Förster expression.^67,68^

Using the point dipole approximation, which can be reasonably assumed given the extremely small magnitude of the transition dipole moment of the solvent (*μ*_H_2_O_ ≈ 5 × 10^−4^ D and *ε*_H_2_O_ ≈ 2 × 10^−4^ cm^−1^ M^−1^ for the 750 nm band, compared with *μ*_F_ = 7.7–17.8 D and *ε* = 0.7–2.8 × 10^5^ cm^−1^ M^−1^ for the used fluorophores, Table S1†), the rate constant for excitation energy transfer by dipolar coupling, *k*_dip_, between a fluorophore and one solvent molecule can be estimated by^69^

5 *k*_dip_ = 1.18 *V*^2^*Θ*

In this expression, *k*_dip_ is in ps^−1^, *Θ* is the overlap integral between the donor emission and the acceptor absorption spectra with their area normalized to unity on the cm^−1^ scale, and *V* is the dipole–dipole coupling energy in cm^−1^. The coupling is given by^69,70^6
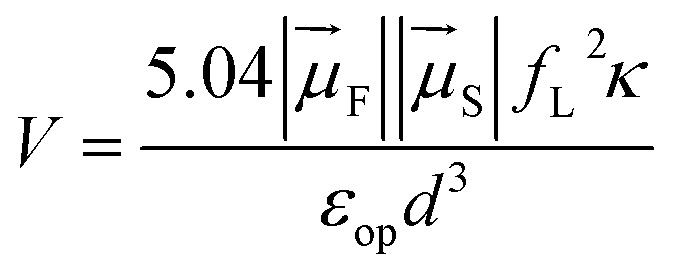
where 
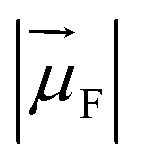
 is the magnitude of the transition dipole of the fluorophore in D, 
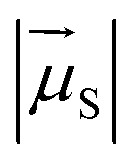
 the magnitude of the transition dipole of a solvent molecule in D, *κ* the orientational factor, *d* the distance between the dipoles in nm, *f*_L_ = (*ε*_op_ + 2)/3 is the Lorentz field correction factor, and *ε*_op_ ≈ *n*^2^ the dielectric constant at optical frequencies, with *n* being the refractive index of the solvent.

Every dye molecule is however not surrounded by one, but by several tens of solvent molecules which can all independently quench its excited state.^29^ The total deactivation, *k*_FRET_, induced by *N* solvent molecules each contributing in average by *k*_dip_ to the excited-state decay is therefore given by

7 *k*_FRET_ = *Nk*_dip_

Using eqn (5)–(7) to estimate the rate of non-radiative deactivation by the solvent requires prior knowledge of the number of solvent molecules contributing to the quenching, as well as their average distance and relative orientation to the solute. Because of the very small transition dipole moment of water, it seems reasonable to assume that only water molecules in the first solvent shell are likely to contribute to quenching the fluorophores. Estimation of the Förster radius (also known as critical quenching radius), *R*_0_, from the spectral overlap integral and the unquenched excited-state lifetime in D_2_O indeed leads to values for red-emitting dyes between 0.2 and 0.3 nm, that is, on the order of the size of one water molecule (Fig. S13, Table S7†). Molecular dynamics (MD) simulations of several fluorophores of different sizes, including ATTO655, ATTO647N, and bimane,^71^ one of the smallest known fluorescent dyes, reveal the size of the first water solvent shell determined from the minimum-distance distribution function to be about 0.45 nm and independent of the nature of the fluorophore (Fig. 5c). The minimum-distance distribution function is calculated with respect to the distance between the centre of mass of the solvent and the closest atom of the solute (see ESI†). It therefore does not correspond to the distance between the transition dipoles of the fluorophore and of the solvent, but provides a lower limit to this quantity.

Increasing the size of the solvent molecules shifts the size of the solvent shell radii to 0.49 nm in methanol and 0.54 nm in ethanol with the probe ATTO655 (Fig. 5d). Using a simple counting procedure, we determined the average number of solvent centres of mass within the first solvent shell for each frame in the simulation. For ATTO655, this number averages to 86.1 ± 3.8 in water (Fig. 5e) and to 50.2 ± 2.6 in methanol (Fig. S14†). For ATTO647N, the number of water molecules in the first solvent shell is 96.5 ± 4.2, while it is 43.6 ± 2.7 for bimane (Fig. S14†), indicating that this number may change at most by a factor up to 2 or 3 between the smallest and the largest investigated probes in our study (all are larger than bimane).

With the results of these MD simulations in hand, we evaluated the contribution of every vibrational overtone component of the H_2_O absorption spectrum to the excited-state decay of a given fluorophore using eqn (5), after determining the magnitude of its transition dipole moment, its overlap integral with the fluorophore emission, and its coupling energy with the fluorophore (eqn (6)). We used a value of *d* = 0.53 nm in water, slightly larger than the minimal solvent shell radius, to take into account the fact that the origin of the transition dipole is located at the centre of the chromophoric unit and not at the edge of the molecule. The orientational factor *κ*^2^ was set to 2/3 after MD simulations confirmed this value for ATTO655 in water and in MeOH, indicating an average random orientation of the many molecules in the first solvent sphere with respect to the dye (see ESI and Fig. S15†). The total calculated dipole–dipole energy transfer rate constant between one dye and one water molecule was then obtained by summing the contributions of the individual bands. Unsurprisingly, the band at 13 050 cm^−1^ (760 nm) displayed the largest contribution to the decay (Fig. S16†). We found values for *k*_dip_, the deactivation rate of the different fluorophores by one water molecule, ranging between 1 × 10^4^ and 7 × 10^6^ s^−1^ which are too low for the process to meaningfully contribute to deactivating the excited state. However, by using eqn (7) and an average value of water molecules *N* = 86 for all fluorophores, we obtained values for *k*_FRET_ large enough to compete with fluorescence emission and which qualitatively very well reproduce the trend of measured quenching rate constant *k*_s_ (Fig. 5f, Table S2†). Assuming identical oscillator strength for methanol and water based on the similarity of their absorption spectra in the 700–850 nm region (Fig. S12†), we were further able to reproduce the quenching rate constant measured in methanol with values of *d* = 0.60 nm and *N* = 50 (Fig. 5g, Table S3†).

The agreement between measured *k*_s_ and calculated *k*_FRET_ is very good at small energy gaps, indicating that quenching of red-emitting fluorophores by the solvent can well be described by resonance energy transfer *via* dipole–dipole coupling. The underlying assumptions to our calculations are: (1) a fixed distance between the fluorophore and the solvent molecules, which seems reasonable given the small size of the solvent and the fact that only the first solvent shell contributes to the quenching; (2) a large number of solvent molecules in the first shell which can all independently act as energy acceptors, which is in agreement with observations for metal ions;^28^ although the number of accepting molecules was fixed here to 86 for H_2_O and 50 for MeOH whereas it actually varies from dye to dye according to the molecular structure, the quantitative effect is negligible on a logarithmic scale as expected variations are of a factor 1.5 at most (between 60 and 120 water molecules per dye); (3) a random orientation of the solvent molecules, which is confirmed by MD simulations. At energy gaps beyond ∼2.1 eV (∼600 nm), the predicted energy transfer rate constant systematically underestimates the measured quenching, which is consistent with other quenching mechanisms dominating the non-radiative decay of the dyes, in particular internal conversion.

Two components control the value of *k*_dip_, namely the dipole–dipole coupling energy and the overlap integral. A closer look at the coupling reveals that its value is essentially constant for all dyes, around 0.6–1.2 cm^−1^ (Fig. S17a†), which is consistent with the weak coupling limit used for the energy gap law and with the fact that no spectral shift is observed in the absorption or in the fluorescence when switching from H_2_O to D_2_O. On the other hand, the spectral overlap integral displays a strong dependence on the energy gap (Fig. S17b†) and is therefore primarily responsible for conferring *k*_s_ its overall exponential dependence on the energy gap, which is a manifestation of the proportionality of the energy transfer rate constant to the overlap integral.^72^ This exponential dependence is also reflected in the FRET efficiency which can be calculated for every dye from *k*_s_ and the unquenched excited-state lifetime in D_2_O or MeOH-d4 and which provides another way of characterizing the measured fluorescence enhancement in perdeuterated solvents (Fig. S18, Table S7†).

The fact that a through-space energy transfer mechanism dominates the excited-state decay of red-emitting fluorophores also provides a rationale for why selective or complete deuteration, fluorination, or alkylation of some rhodamines and oxazines led to only a moderate enhancement of their fluorescence quantum yield or lifetime:^73–76^ intermolecular quenching by high-energy O–H vibrations of the solvent is more efficient a process than intramolecular internal conversion *via* C–H or N–H vibrations, as illustrated by the larger attenuation factor observed in our energy gap law analysis. The importance of the intermolecular interaction with the solvent for the quenching was stressed in a study with several oxazines for which the excited-state lifetime increased much more in deuterated solution than in the gas phase with deuterated dyes.^75^

It should further be noted that, in addition to O–H bonds, also C–H bonds seem to be able to weakly quench excited fluorophores not only intramolecularly,^73–76^ but also when they are present in the solvent molecules: selective deuteration of the hydrogens on the hydrocarbon chain of alcohols led to a small increase of the fluorescence quantum yield and lifetime of the oxazine ATTO655 (Fig. 3c), and a small solvent-assisted quenching rate constant is measurable in MeOD or EtOD (Fig. 3d). The observed isotope effect on the quenching is however much smaller for C–H (1.4–2.5) than for O–H bonds (4–7). One may hypothesize that the lower fundamental vibrational frequency of C–H vibrations (∼3000 cm^−1^) compared to O–H vibrations (∼3400 cm^−1^) makes it less likely for a suitable overtone or combination band with a strong enough absorption coefficient to be in resonance with the emission of the fluorophore.^30^ Furthermore, hydrogen bonds between the solvent molecules lead to a strong anharmonic potential for O–H vibrations, making the transitions to overtones and combination vibrations more likely than for C–H vibrations.^58^ However, other unknown quenching mechanisms cannot be excluded, as it was noted that also OD groups in D_2_O may still act as weak quenchers (the fluorescence of ATTO655 is not fully restored in D_2_O), and the existence of completely different solvent-assisted quenching mechanisms such as, for example, reversible covalent interactions with H atoms in the excited state of dyes have been suggested.^19,20^

For practical applications in which the brightness of fluorescent dyes is to be improved, it seems nonetheless that preventing quenching by O–H vibrations should be the most efficient strategy. As the observed isotope effects on the fluorescence quantum yield and lifetime strongly depend on the energy gap, protecting fluorophores from solvent OH groups seems particularly useful for red-emitting dyes,^25,26^ for which fluorescence enhancements of up to 3.1 and FRET efficiencies to the solvent of up to 67% could be observed. Similarly large isotope effects on the fluorescence quantum yield and lifetime were reported with other fluorophores in water^4^ and in other solvents^19,37^ for probes already possessing a fluorescence quantum yield in the protonated solvent of at least a few percent. One should note that much stronger fluorescence enhancements can however be reached with fluorophores for which the quenching mechanism involves hydrogen bonding and/or proton transfer leading to ultrafast deactivation of the excited state.^39,40^

## Conclusions

**Fig. 6:**
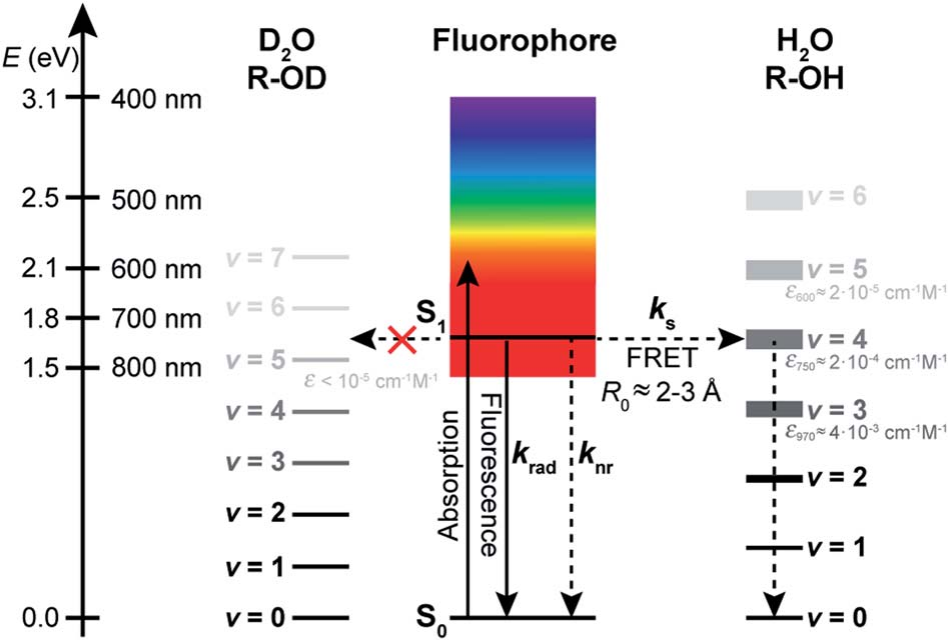
Schematic representation of the proposed mechanism leading to solvent-assisted quenching of red-emitting fluorophores by resonant energy transfer to vibrational overtones and combination transitions of O–H vibrations in H_2_O and alcohols (R-OH). The same process is inefficient in D_2_O or perdeuterated alcohols (R-OD) because of their lack of absorption in the 700–900 nm region.

By investigating the fluorescence properties of 42 fluorophores belonging to 5 common dye classes, we found that solvents bearing functional groups with high-energy vibrations such as OH moieties can act as efficient fluorescence quenchers, in particular if the fluorophores absorb and emit beyond 600 nm. The quenching efficiency for these dyes does not depend on H-bonds with the solvent, but rather proceeds through space by resonant energy transfer from the electronically excited dye to combination bands of O–H vibrations in the (highly concentrated) solvent which spectrally overlap with the dye fluorescence (Fig. 6). The latter can be restored in perdeuterated analogues of the solvents or in non-alcoholic organic solvents which, in the absence of OH groups, do not absorb in the 700–900 nm spectral region.

Resonant energy transfer from the electronic state of an organic molecule to vibrational overtones of the solvent has been hypothesized before to explain the quenching of the phosphorescence of some organic molecules,^42^ but we were able here for the first time to demonstrate the generality of this process for the fluorescence of large organic dyes in water and methanol. This type of quenching is expected to take place with any fluorophore absorbing and emitting in the 600–900 nm spectral region, and is only observed in the absence of faster competing processes. It might also become noticeable for probes absorbing below 600 nm and the excited-state of which is long-lived, as in trianguleniums (15–20 ns)^13^ or 2,3-diazabicyclo[2.2.2]oct-2-ene (300–500 ns).^15^

Our results suggest that the quencher solvent molecules act independently of each other, which opens the door to using standard organic fluorophores to count solvent molecules in their direct surroundings like it has been done with lanthanide ions.^30^ Such molecular counters based on organic fluorophores could however easily be applied in key biological environments. Furthermore, understanding the molecular basis of this universal quenching mechanism opens up new perspectives for the design of brighter molecules and materials by avoiding solvents possessing high-energy vibrations or by explicitly shielding them from solvent molecules, for example *via* encapsulation into cyclodextrins,^77–80^ cucurbiturils,^80–86^ or zeolites.^87,88^

## Abbreviations

AF: Alexa Fluor
5CR6G: 5-carboxyrhodamine 6G
TMR: Tetramethylrhodamine
ICG: Indocyanine green

## Conflicts of interest

There are no conflicts to declare.

## Supplementary Material

SC-012-D0SC05431C-s001

## References

[cit1] Moerner W. E., Fromm D. P. (2003). Rev. Sci. Instrum..

[cit2] Lavis L. D., Raines R. T. (2014). ACS Chem. Biol..

[cit3] Fürstenberg A. (2017). Chimia.

[cit4] Stryer L. (1966). J. Am. Chem. Soc..

[cit5] Kropp J. L., Windsor M. W. (1963). J. Chem. Phys..

[cit6] Kropp J. L., Windsor M. W. (1965). J. Chem. Phys..

[cit7] Sens R., Drexhage K. H. (1981). J. Lumin..

[cit8] Inoue H., Hida M., Nakashima N., Yoshihara K. (1982). J. Phys. Chem..

[cit9] Joshi H. C., Gooijer C., van der Zwan G. (2002). J. Phys. Chem. A.

[cit10] Fürstenberg A., Vauthey E. (2005). Photochem. Photobiol. Sci..

[cit11] Sailer B. L., Nastasi A. J., Valdez J. G., Steinkamp J. A., Crissman H. A. (1997). J. Histochem. Cytochem..

[cit12] Beeby A., Clarkson I. M., Dickins R. S., Faulkner S., Parker D., Royle L., de Sousa A. S., Williams J. A. G., Woods M. (1999). J. Chem. Soc., Perkin Trans. 2.

[cit13] Bisballe N., Laursen B. W. (2020). Chem.–Eur. J..

[cit14] Magde D., Rojas G. E., Seybold P. G. (1999). Photochem. Photobiol..

[cit15] Nau W. M., Zhang X. (1999). J. Am. Chem. Soc..

[cit16] Merkel P. B., Kearns D. R. (1972). J. Am. Chem. Soc..

[cit17] Van Houten J., Watts R. J. (1975). J. Am. Chem. Soc..

[cit18] Gabriel S., Elhanan W. (1976). Z. Phys. Chem..

[cit19] Nau W. M., Adam W., Scaiano J. C. (1996). Chem. Phys. Lett..

[cit20] Nau W. M., Greiner G., Rau H., Wall J., Olivucci M., Scaiano J. C. (1999). J. Phys. Chem. A.

[cit21] Schmidt R., Bodesheim M. (1994). J. Phys. Chem..

[cit22] Wang B., Ogilby P. R. (1993). J. Phys. Chem..

[cit23] Hurst J. R., Schuster G. B. (1983). J. Am. Chem. Soc..

[cit24] Mirbach M. J., Mirbach M. F., Cherry W. R., Turro N. J., Engel P. (1978). Chem. Phys. Lett..

[cit25] Lee S. F., Vérolet Q., Fürstenberg A. (2013). Angew. Chem., Int. Ed..

[cit26] Klehs K., Spahn C., Endesfelder U., Lee S. F., Fürstenberg A., Heilemann M. (2014). ChemPhysChem.

[cit27] Robinson G. W., Frosch R. P. (1963). J. Chem. Phys..

[cit28] Haas Y., Stein G. (1971). J. Phys. Chem..

[cit29] Haas Y., Stein G. (1971). J. Phys. Chem..

[cit30] Horrocks W. D., Sudnick D. R. (1979). J. Am. Chem. Soc..

[cit31] Faulkner S., Pope S. J. A., Burton-Pye B. P. (2005). Appl. Spectrosc. Rev..

[cit32] Dobretsov G. E., Syrejschikova T. I., Smolina N. V. (2014). Biophysics.

[cit33] Cohen B., Huppert D. (2001). J. Phys. Chem. A.

[cit34] Agmon N. (2005). J. Phys. Chem. A.

[cit35] Moore R. A., Lee J., Robinson G. W. (1985). J. Phys. Chem..

[cit36] Fita P., Fedoseeva M., Vauthey E. (2011). Langmuir.

[cit37] Flom S. R., Barbara P. F. (1985). J. Phys. Chem..

[cit38] Yatsuhashi T., Inoue H. (1997). J. Phys. Chem. A.

[cit39] Fita P., Fedoseeva M., Vauthey E. (2011). J. Phys. Chem. A.

[cit40] Dereka B., Vauthey E. (2017). Chem. Sci..

[cit41] Biczók L., Bérces T., Inoue H. (1999). J. Phys. Chem. A.

[cit42] Ermolaev V. L., Sveshnikova E. B. (1973). Chem. Phys. Lett..

[cit43] Ermolaev V. L., Sveshnikova E. B., Bodunov E. N. (1996). Physics-Uspekhi.

[cit44] Ermolaev V. L., Sveshnikova E. B. (1979). J. Lumin..

[cit45] Siebrand W. (1967). J. Chem. Phys..

[cit46] Lakowicz J. R. , Principles of Fluorescence Spectroscopy, Kluwer Academic, New York, 2nd edn, 1999

[cit47] Van S.-P., Hammond G. S. (1978). J. Am. Chem. Soc..

[cit48] Murov S. L. , Carmichael I. and Hug G. L., Handbook of Photochemistry, Marcel Dekker, Inc., New York, 1993

[cit49] Strickler S. J., Berg R. A. (1962). J. Chem. Phys..

[cit50] Clark T., Heske J., Kühne T. D. (2019). ChemPhysChem.

[cit51] Taft R. W., Kamlet M. J. (1976). J. Am. Chem. Soc..

[cit52] Marcus Y. (1993). Chem. Soc. Rev..

[cit53] Stennett E. M. S., Ciuba M. A., Levitus M. (2014). Chem. Soc. Rev..

[cit54] Englman R., Jortner J. (1970). Mol. Phys..

[cit55] Tétreault N., Muthyala R. S., Liu R. S. H., Steer R. P. (1999). J. Phys. Chem. A.

[cit56] Chynwat V., Frank H. A. (1995). Chem. Phys..

[cit57] Siebrand W. (1967). J. Chem. Phys..

[cit58] Braun C. L., Smirnov S. N. (1993). J. Chem. Educ..

[cit59] Pope R. M., Fry E.
S. (1997). Appl. Opt..

[cit60] Litjens R. A. J., Quickenden T. I., Freeman C. G. (1999). Appl. Opt..

[cit61] Morita H., Nagakura S. (1974). J. Mol. Spectrosc..

[cit62] Lange K. R., Wells N. P., Plegge K. S., Phillips J. A. (2001). J. Phys. Chem. A.

[cit63] Waggener W. C. (1958). Anal. Chem..

[cit64] Timmer R. L. A., Tielrooij K. J., Bakker H. J. (2010). J. Chem. Phys..

[cit65] Aharoni A., Oron D., Banin U., Rabani E., Jortner J. (2008). Phys. Rev. Lett..

[cit66] Thorning F., Jensen F., Ogilby P. R. (2020). J. Phys. Chem. B.

[cit67] Förster T. (1948). Ann. Phys..

[cit68] Dexter D. L. (1953). J. Chem. Phys..

[cit69] Pullerits T., Hess S., Herek J. L., Sundstrom V. (1997). J. Phys. Chem. B.

[cit70] Fürstenberg A., Julliard M. D., Deligeorgiev T. G., Gadjev N. I., Vasilev A. A., Vauthey E. (2006). J. Am. Chem. Soc..

[cit71] Kosower E. M., Pazhenchevsky B., Hershkowitz E. (1978). J. Am. Chem. Soc..

[cit72] Haugland R. P., Yguerabide J., Stryer L. (1969). Proc. Natl. Acad. Sci. U. S. A..

[cit73] Kolmakov K., Belov V. N., Bierwagen J., Ringemann C., Mueller V., Eggeling C., Hell S. W. (2010). Chem. – Eur. J..

[cit74] Wurm C., Kolmakov K., Gottfert F., Ta H., Bossi M., Schill H., Berning S., Jakobs S., Donnert G., Belov V., Hell S. (2012). Opt. Nanoscopy.

[cit75] Kusinski M., Nagesh J., Gladkikh M., Izmaylov A. F., Jockusch R. A. (2019). Phys. Chem. Chem. Phys..

[cit76] Ferreira J. A. B., Costa S. M. B. (2006). Chem. Phys..

[cit77] Buston J. E. H., Young J. R., Anderson H. L. (2000). Chem. Commun..

[cit78] Yau C. M. S., Pascu S. I., Odom S. A., Warren J. E., Klotz E. J. F., Frampton M. J., Williams C. C., Coropceanu V., Kuimova M. K., Phillips D., Barlow S., Brédas J.-L., Marder S. R., Millar V., Anderson H. L. (2008). Chem. Commun..

[cit79] Arunkumar E., Forbes C. C., Smith B. D. (2005). Eur. J. Org. Chem..

[cit80] Dsouza R. N., Pischel U., Nau W. M. (2011). Chem. Rev..

[cit81] Bhasikuttan A. C., Mohanty J., Nau W. M., Pal H. (2007). Angew. Chem., Int. Ed..

[cit82] Jiang T., Qu G., Wang J., Ma X., Tian H. (2020). Chem. Sci..

[cit83] Koner A. L., Nau W. M. (2007). Supramol. Chem..

[cit84] Mohanty J., Nau W. M. (2005). Angew. Chem., Int. Ed..

[cit85] Nau W. M., Hennig A., Koner A. L. (2008). Springer Ser. Fluoresc..

[cit86] Nau W. M., Mohanty J. (2005). Int. J. Photoenergy.

[cit87] Calzaferri G., Huber S., Maas H., Minkowski C. (2003). Angew. Chem., Int. Ed..

[cit88] Nicolet O., Huber S., Lovey C., Chappellet S., Perrenoud J., Pauchard M., Ferrini R., Zuppiroli L. (2009). Adv. Funct. Mater..

[cit89] Bodunov E. N., Danilov V. V., Panfutova A. S., Simões Gamboa A. L. (2016). Ann. Phys..

