# Supplementary material for: Universal quenching of common fluorescent probes by water and alcohols
Source: Chem Sci. 2020 Nov 19;12(4):1352–62. doi: 10.1039/d0sc05431c (PMC8179231; doi:10.1039/d0sc05431c)
Supplement: SC-012-D0SC05431C-s001 [file SC-012-D0SC05431C-s001.pdf]

Electronic Supplementary Material (ESI) for Chemical Science.
This journal is © The Royal Society of Chemistry 2020

Electronic Supplementary Information

Universal quenching of common fluorescent probes by water and alcohols

Jimmy Maillard,[‡,a,b] Kathrin Klehs,[‡,c] Christopher A. Rumble,[a] Eric Vauthey,[a] Mike Heilemann,[*,c] and Alexandre Fürstenberg[*,a,b]

[a] Department of Physical Chemistry and [b] Department of Inorganic and Analytical Chemistry, University of Geneva, 1211 Geneva, Switzerland

[c] Institute for Physical and Theoretical Chemistry, Goethe University Frankfurt, 60489 Frankfurt am Main, Germany

[‡] These authors contributed equally

heilemann@chemie.uni-frankfurt.de, alexandre.fuerstenberg@unige.ch

Contents

1. Experimental section page 2

2. Photophysical data analysis 4

3. Decomposition of the H_2O absorption spectrum 6

4. FRET analysis 6

5. MD simulations 9

6. Supplementary tables 11

7. Supplementary figures 17

8. Supplementary references 31

1. Experimental section

Materials. All dyes used in this study were obtained as N-hydroxysuccinimidyl (NHS) esters unless stated otherwise. We purchased Alexa Fluors (AF) AF488, AF514, AF532, AF546, AF568, AF594, AF610x, AF633, AF647, AF680, AF700, 5-carboxyrhodamine 6G (5CR6G), and tetramethylrhodamine 5-maleimide (TMR) from Life Technologies, ATTO488, ATTO495, ATTO520, ATTO565, ATTO594, ATTO633, ATTO647N, ATTO655, ATTO655 carboxylic acid, ATTO680, ATTO680 carboxylic acid, ATTO700, ATTO700 carboxylic acid, ATTO725, and ATTO740 from ATTO-Tec, DY505, DY530, DY560, DY590, DY649P1, DY682, DY731, DY749P1, DY778, and DY800 from Dyomics, Cy3B, Cy3.5, Cy5, Cy5.5, and Cy7 from GE Healthcare, and indocyanine green (ICG, no reactive functionalization) from Sigma-Aldrich. NHS esters were assumed to fully hydrolyse to a carboxyl moiety in aqueous solution, and for the dyes for which we had the carboxylic acid and the NHS ester, no differences in their photophysics could be observed. The reference dyes were received in laser dye quality. Fluorescein-27 (F27), rhodamine 101 (R101), and oxazine 1 perchlorate (Ox1) were from Radiant Dyes, rhodamine 6G (R6G) from Lambda Physik and 1,1'3,3,3',3'-hexamethylindotricarbocyanine iodide (HITCI) (98.8%) from Sigma-Aldrich. We thank Prof. K. H. Drexhage (Universität-Gesamthochschule Siegen) for providing the oxazine derivative MR121 maleimide.

All organic solvents were of analytical or higher grade and used without further purification, except acetonitrile which was freshly distilled before use. H_2O was purified using a Milli-Q system (Millipore) or an Arium basic filtration system (Sartorius). D_2O (>99.8 % D atom content) was purchased from Armar Chemicals or from Euriso-Top, methanol-d1 from Sigma-Aldrich, methanol-d3 from Armar Chemicals, methanol-d4 and hexafluoroisopropanol (HFIP) from Apollo Scientific, and all other deuterated solvents from Cambridge Isotope Laboratories.

Steady-state photophysics. Absorption spectra were recorded on a Jasco V-650 UV/Vis or a Cary 50 spectrophotometer using blank correction with the respective solvent. Fluorescence spectra were recorded on a FP-8500 (Jasco) or a FluoroMax-4 (Horiba Jobin Yvon, equipped with a R2658 detector from Hamamatsu) fluorescence spectrometer and corrected for the wavelength dependence of the detector. All spectra were measured in either quartz or disposable acrylic 1-cm optical pathlength cuvettes.

Fluorescence quantum yields of all fluorophores in H_2O and D_2O were determined from integrated fluorescence spectra relative to a respective reference dye acquired under the same experimental conditions. Fluorescence quantum yield measurements of the dyes in other

solvents were carried out using the relative method by integration of the fluorescence spectra and using the given fluorophore in H_2O as a relative standard. Reference dyes were used as received without further purification, dissolved at millimolar concentration in 0.1 M NaOH (F27), absolute ethanol (R6G, R101, Ox1), H_2O (R6G), or DMSO (HITCI), and stored at 4 °C. Stock solutions of the synthetic target dyes at 0.5-10 mM concentration were prepared in anhydrous DMF or DMSO and stored at -20 °C until used. Target fluorophores and reference dyes were freshly diluted into the solvent of interest directly before the measurement. Concentrations were adjusted to similar absorbance values at the excitation wavelength, and the absorbance values of the absorption band corresponding to the S_0–S_1 transition were kept below 0.1. All measurements were carried out in air-saturated solutions.

Fluorescence quantum yields (Φ_{fl}) of the target fluorophores were calculated according to the relationship[S1]

$$\Phi_{\mathrm{fl},x} = \Phi_{\mathrm{fl},s} \frac{B_r(\lambda_{\mathrm{ex}})}{B_x(\lambda_{\mathrm{ex}})} \frac{\int F_x(\lambda)\mathrm{d}\lambda}{\int F_r(\lambda)\mathrm{d}\lambda} \frac{n_x^2}{n_r^2} \qquad \text{(S1)}$$

In this equation, the subscripts x and s indicate the target fluorophore and the reference dye, respectively, $F(\lambda)$ denotes the wavelength-dependent intensity of the corrected fluorescence spectrum which is integrated over the whole emission range, and n is the refractive index of the solvent. The term $B(\lambda_{ex})$ represents the fraction of incident light absorbed by the sample and is given by

$$B(\lambda_{\mathrm{ex}}) = 1 - 10^{-A(\lambda_{\mathrm{ex}})} \qquad \text{(S2)}$$

where $A(\lambda_{ex})$ is the absorbance of the sample at the excitation wavelength.

Fluorescence lifetime measurements. Fluorescence lifetime measurements were performed using the time-correlated single-photon counting (TCSPC) technique under magic angle conditions.[S2] Lifetimes in H_2O and D_2O were measured using excitation at 470 nm (PicoQuant LDH-P-C-470 pulsed laser diode) or 635 nm (PicoQuant LDH-P-C-635B). Excitation light in the fluorescence pathway was removed using a 500 nm (Semrock BLP01-488R-25) or a 650 nm (Semrock BLP01-635R-25) long-pass filter. The instrument response function (IRF) was around 210 ps with 470 nm excitation and 660 ps with 635 nm excitation. Alternatively, fluorescence lifetime measurements were performed on a setup using a tuneable pulsed picosecond excitation source (NKT Photonics SuperK EXTREME). The fluorescence

emission from the sample was collected into an optical fibre, spectrally filtered using a spectrograph (Horiba Triax 190) and detected on a photomultiplier tube (PicoQuant PMA 192C). The wavelength-dependent time resolution of this instrument was 170-200 ps. Both setups agree on the measured excited-state lifetimes within the experimental uncertainty.

All fluorescence lifetimes were extracted from the measured traces by iterative reconvolution of a trial function (single exponential or sum of exponential functions when required) with the measured IRF. In the case of multiexponential decays (Cy dyes),[S3] the average lifetime was calculated from the amplitude average of the exponential components.[S4] The uncertainty on fluorescence lifetimes is estimated to ± 0.05 ns.

2. Photophysical data analysis

Standard photophysical relationships were used to determine the radiative rate (k_{rad}) and the sum of non-radiative (k_{nr}) and solvent-assisted quenching rate (k_s) from the fluorescence quantum yield (Φ_{fl}) and the excited-state lifetime (τ_{S1}). The rate constant for the depopulation of the excited state of a standard organic dye (k_{S1}) in a pure solution may be written as the sum of radiative and nonradiative deactivation processes:

$$k_{S1} = \left(\tau_{S1}\right)^{-1} = k_{rad} + k_{nr} + k_s \qquad \text{(S3)}$$

where $k_{rad} = \Phi_{fl} \cdot k_{S1}$ is the radiative rate constant, k_{nr} a first-order nonradiative decay rate constant taking into account all intrinsic nonradiative decay processes (internal conversion, intersystem crossing, etc.), and k_s a rate constant representing the nonradiative decay selectively induced by the solvent. In order to determine the solvent quenching rate constant k_s for ATTO655 in different solvents, we first calculated k_{rad} in all solvents from the fluorescence quantum yield and excited-state lifetime, and postulated that $k_s = 0$ in acetonitrile-d3, the solvent in which the longest excited-state lifetime was measured. With this we obtained a value of $k_{nr} = 6.8(8) \cdot 10^7$ s^{-1} which we assumed constant in all solvents in order to calculate k_s.

For all other dyes, k_s was determined from the excited-state lifetimes as follows. Under the reasonable assumptions that (1) k_{rad} is independent of the solvent for these well-behaved dyes, and that (2) k_{nr} does not significantly change between a given protonated solvent (S_H, *e.g.* H_2O) and its deuterated analogue (S_D, *e.g.* D_2O), the difference in excited-state decay rate constants

between a protonated and a deuterated solvent directly yields the quenching rate constant for this solvent, within the limit that quenching by the deuterated solvent is negligible:

$$k_{S1}(S_H) - k_{S1}(S_D) = \Delta k_{S1} = k_s(S_H) - k_s(S_D) \approx k_s(S_H) \quad \text{(S4)}$$

Instead of measuring the photophysical properties of the dye solely in pure solvents, one can also treat the protonated solvent as a quencher which can be added in different amounts to a solution of fluorophore in a deuterated solvent. In the case of purely dynamic quenching, the excited-state decay rate constant of a fluorophore F depends on the concentration of protonated solvent (the quencher) and can be expressed as

$$k_{S1} = (\tau_{S1})^{-1} = k_{rad} + k_{nr} + k_q \cdot [S_H] \quad \text{(S5)}$$

where k_q is the bimolecular quenching rate constant for the reaction

$$F^{*,el} + S_H \longrightarrow F + S_H^{*,vib} \quad \text{(S6)}$$

In the absence of quencher, the observed decay rate is simply

$$(k_{S1})_0 = (\tau_{S1})_0^{-1} = (\tau_0)^{-1} = k_{rad} + k_{nr} \quad \text{(S7)}$$

Dividing eq. S5 by eq. S7 yields the Stern-Volmer expression for dynamic quenching:[S5]

$$\frac{k_{S1}}{(k_{S1})_0} = \frac{\tau_0}{\tau_{S1}} = \frac{k_{rad} + k_{nr} + k_q[S_H]}{k_{rad} + k_{nr}} = 1 + k_q\tau_0[S_H] \quad \text{(S8)}$$

By comparing equations S3 and S5, one concludes that

$$k_s = k_q[S_H] \quad \text{(S9)}$$

This relationship has been experimentally verified in the present work for the dyes for which a Stern-Volmer titration was performed.

3. Decomposition of the H_2O absorption spectrum

Because of interference patterns arising in the signal when using an empty reference cuvette, the absorption spectrum of pure H_2O and pure MeOH was obtained using D_2O as a reference. Neither D_2O nor MeOH-d4 absorb in any measurable manner over a path length of 1 cm in the investigated spectral region.[S6, 7] We further made sure that the measured value for the molar absorption coefficient matched known literature values ($\varepsilon_{700} \approx 4 \cdot 10^{-5}$ cm^{-1}·M^{-1} for H_2O and $\varepsilon_{750} \approx 3 \cdot 10^{-4}$ cm^{-1}·M^{-1} for MeOH).[S7, 8] The spectrum of H_2O was then decomposed on the wavenumber scale using a sum of 6 Gaussian functions and of a baseline component (λ^{-4} dependence) using a custom written Matlab script. Inclusion of all 7 components was required in order to reproduce the data. The centres of the Gaussian functions were fixed at or close to known overtones and/or combination modes of the fundamental vibrational frequencies ν_1 (symmetric stretching), ν_2 (bending), and ν_3 (asymmetric stretching). The central frequencies of the Gaussians and their assignment are listed in Table S6.

4. FRET analysis

Transition dipole moment magnitudes were estimated from the relationship

$$|\vec{\mu}| = \sqrt{\frac{3he^2 f}{8\pi^2 m_e \nu_0}} \qquad \text{(S10)}$$

where h is Planck's constant, e the charge of the electron, m_e the mass of the electron, and ν_0 the central transition frequency in s^{-1}. In this equation, f is the oscillator strength which is obtained by integrating over the absorption band of interest on the wavenumber ($\bar{\nu}$) scale (in cm^{-1}) using the following relationship:[S9]

$$f = 4.3 \cdot 10^{-9} \int \varepsilon(\bar{\nu}) \mathrm{d}\bar{\nu} \qquad \text{(S11)}$$

The overlap integrals (Θ) between the fluorophore emission and the solvent absorption were computed on the wavenumber scale using area-normalized spectra as required by eq. 5 of the main text. An integral Θ_i was calculated for every overlap between the emission spectrum of the dye and the ith component of the water absorption spectrum. In parallel, the coupling, V_i,

between a fluorophore and the ith absorption band of one water molecule was estimated using the transition dipole magnitude for the ith absorption band:

$$V_i = \frac{5.04 \cdot |\vec{\mu}_F| |\vec{\mu}_{S,i}| f_L^2 \kappa}{\varepsilon_{op} d^3} \quad \text{(S12)}$$

where V is expressed in cm^{-1}, μ in D, and d in nm.

From the coupling and the overlap integral for the ith absorption of water, the rate constant for excitation energy transfer by dipolar coupling between a fluorophore and the ith absorption band of one water molecule, $k_{dip,i}$, could be determined (Figure S16):

$$k_{dip,i} = 1.18 \cdot V_i^2 \cdot \Theta_i \quad \text{(S13)}$$

where k_{dip} is expressed in ps^{-1}, V in cm^{-1}, and Θ in cm.

The total energy transfer rate constant between a fluorophore and one water molecule was obtained by summing over all water absorption bands:

$$k_{dip} = \sum_i k_{dip,i} \quad \text{(S14)}$$

The total dipole-dipole resonance energy transfer rate constant, k_{FRET}, was obtained by multiplying k_{dip} by the total number of solvent molecules N (main text, eq. 7). For the sake of this computation the result of which is represented on a semi-log scale, N was kept constant (N = 86 in water, N = 50 in methanol, see main text).

The total coupling and total overlap integral (Figure S17) were estimated by summing over all i components of the water absorption spectrum:

$$V = \sum_i V_i \quad \text{(S15)}$$

$$\Theta = \sum_i \Theta_i \quad \text{(S16)}$$

The energy transfer rate constant, k_{FRET}, is also defined in Förster's theory as:

$$k_{\mathrm{FRET}} = k_{\mathrm{D}}\left(\frac{R_0}{d}\right)^6 = \frac{1}{\tau_{\mathrm{D}}^0}\left(\frac{R_0}{d}\right)^6 \qquad \text{(S17)}$$

where $k_{\mathrm{D}} = 1/\tau^0_{\mathrm{D}}$ is the decay rate constant of the excited donor fluorophore in the absence of energy transfer, d is the distance, and R_0 is the Förster radius or critical quenching radius defined as the distance at which the energy transfer rate constant, k_{FRET}, and the decay rate constant of the excited donor in the absence of energy transfer, k_{D}, are equal.[S10] R_0 can be evaluated from the relationship[S10]

$$R_0 = 0.02108 \cdot \left(\frac{\kappa^2 \Phi_{\mathrm{D}}}{n^4} \cdot J_\lambda\right)^{1/6} \qquad \text{(S18)}$$

where Φ_{D} is the quantum yield of the fluorophore in the absence of energy transfer and J_λ is the spectral overlap integral expressed in units of $\mathrm{M^{-1}\cdot cm^{-1}\cdot nm^4}$, with

$$J_\lambda = \int F_{\mathrm{D}}(\lambda)\varepsilon_{\mathrm{A}}(\lambda)\lambda^4\, \mathrm{d}\lambda \quad \text{with} \int F_{\mathrm{D}}(\lambda)\mathrm{d}\lambda = 1 \qquad \text{(S19)}$$

In equation S19, F_{D} is the area-normalized fluorescence spectrum of the donor fluorophore. The values of R_0 in Table S7 and Figure S13 were obtained by using $\Phi_{\mathrm{D}} = \Phi_{\mathrm{fl}}(D_2O)$, $\kappa^2 = 2/3$, and $n = 1.332$ as the index of refraction for H_2O.

The energy transfer efficiency, Φ_{FRET}, is directly related to k_{FRET} and given by

$$\Phi_{\mathrm{FRET}} = \frac{k_{\mathrm{FRET}}}{k_{\mathrm{D}} + k_{\mathrm{FRET}}} \qquad \text{(S20)}$$

In the present case, k_{FRET} was experimentally determined in water as $k_{\mathrm{s}} = k_{\mathrm{S1}}(H_2O) - k_{\mathrm{S1}}(D_2O)$, and under the same assumption $k_{\mathrm{D}} = k_{\mathrm{S1}}(D_2O)$. Therefore, equation S20 can be rewritten as:

$$\Phi_{\mathrm{FRET}}(H_2O) = \frac{k_{\mathrm{s}}(H_2O)}{k_{\mathrm{S1}}(D_2O) + k_{\mathrm{s}}(H_2O)} = \frac{k_{\mathrm{s}}(H_2O)}{k_{\mathrm{S1}}(H_2O)} = k_{\mathrm{s}}(H_2O)\cdot \tau_{\mathrm{S1}}(H_2O) \qquad \text{(S21)}$$

By analogy, the following relationship holds true in methanol:

$$\Phi_{\mathrm{FRET}}(\mathrm{MeOH}) = \frac{k_{\mathrm{s}}(\mathrm{MeOH})}{k_{\mathrm{S1}}(\mathrm{MeOH\text{-}d4}) + k_{\mathrm{s}}(\mathrm{MeOH})} = \frac{k_{\mathrm{s}}(\mathrm{MeOH})}{k_{\mathrm{S1}}(\mathrm{MeOH})} \qquad \text{(S22)}$$

Equations S21 and S22 were used to evaluate the energy transfer efficiency in Table S7 and Figure S18.

5. MD simulations

MD simulations of fluorophores in water were performed using GROMACS 2018.1[S11] and the AMBER03 forcefield. Point charges for atoms in the solute were determined from RESP fitting of the electrostatic potential from quantum calculations performed using Gaussian 09[S12] with the B3LYP functional and a 6-311G** basis set. The *antechamber*[S13] and *acpype*[S14] programs were used to generate topologies for the probes. The TIP4P model was used to simulate water,[S15] and standard AMBER03 parameters were used for MeOH and EtOH.

Each solute-solvent system was simulated using 1 chromophore molecule in a cubic box with dimensions of 6 × 6 × 6 nm. The simulation boxes were generated by placing the chromophore at the centre of the box and adding water using the *gmx solvate* command or *gmx insert-molecules* for the other solvents. There were ~7100 water molecules, 3305 MeOH, and 2273 EtOH molecules in each solvent system.

Integration was carried out using the Verlet leap-frog algorithm with a step size of 2 fs. Non-bonded interactions were calculated using a Verlet neighbour list with a cutoff radius of 14 Å. Long-range electrostatics were calculated using the particle-mesh Ewald method.[S16] Hydrogen-containing bonds were constrained using the LINCS algorithm.[S17] The temperature was 293.15 K and was controlled using the modified Berendsen thermostat[S18] with a relaxation time of 0.5 ps.

Equilibration was accomplished using the following procedure. First, an energy minimization procedure was carried out using the steepest-descent method with maximum force stopping point of 500 $kJ \cdot mol^{-1} \cdot nm^{-1}$. Next, three consecutive 500 ps simulations were carried out in the NVT, NPT, and NVT ensembles. The pressure in the NPT simulation was set to 1.013 bar and regulated using the Berendsen barostat with a relaxation time of 5 ps. Following equilibration, a 5 ns production simulation was carried out in the NVT ensemble and coordinates were saved at 1 ps intervals, giving 5000 configurations for later analysis.

Minimum-distance distribution functions, $g(r_{min})$, were then calculated according to standard literature procedures.[S19, 20] These distribution functions are calculated with respect to the

distance between the solvent centre of mass and the closest atom of the solute. Normalization was accomplished using a Monte Carlo procedure that generated randomly distributed centres of mass from which minimum-distance distributions were calculated. Solute structures for the normalization procedure were taken from the production simulations, and the number of randomly generated centres of mass per frame was the same as the number of solvent molecules in the simulation.

The average FRET orientation factor, $\langle\kappa^2\rangle$, for energy transfer between ATTO655 and water was estimated by averaging individual κ^2 values for all water molecules within 0.45 nm, the radius of the first solvent shell as determined by $g(r_{\text{min}})$. The orientation factor for the ith solvent molecule and the average are calculated according to:

$$\kappa_i^2 = \left[\vec{D}\cdot\vec{A} - 3\left(\vec{D}\cdot\vec{R}\right)\left(\vec{A}\cdot\vec{R}\right)\right]^2$$
$$\left\langle\kappa^2\right\rangle = \frac{1}{N}\sum_{i=1}^{N}\kappa_i^2 \qquad \text{(S17)}$$

In these equations, N is the total number of first solvent shell waters in the 5000 saved simulation frames, and the vectors $\vec{D}$ and $\vec{A}$ are unit vectors lying along the transition dipole moments for the FRET donor (ATTO655) and acceptor (water) molecules. For ease of computation, $\vec{D}$ was taken to be a vector connecting the ATTO655 central N and O atoms of the oxazine core, and $\vec{A}$ connects the two hydrogens of water. Finally, $\vec{R}$ is a unit vector connecting the centres of mass of ATTO655 and the water molecule in question. These vectors are illustrated in Figure S15. We find that $\langle\kappa^2\rangle = 0.66$ with a standard deviation of 0.70. This average corresponds well with the isotropic prediction, which is not surprising given the large number of water molecules and the lack of strong specific interactions between ATTO655 and water. We performed the same evaluation for MeOH following the same procedure and using the O and H atoms of the OH group to define the vector $\vec{A}$.

6. Supplementary Tables

Table S1. Steady-state photophysical properties of the investigated fluorophores in water and dye class to which they belong: absorption (λ_{abs}) and emission (λ_{em}) maxima, S_0-S_1 energy gap in eV (ΔE_{00}) and in nm (λ_{00}), molar decadic absorption coefficient at the absorption maximum provided by the manufacturer (ε), and calculated S_0-S_1 transition dipole moment magnitude μ_F. The dyes are listed by decreasing ΔE_{00}.

Dye	λ_{abs} (nm)	λ_{em} (nm)	ΔE_{00} (eV)	λ_{00} (nm)	ε ($cm^{-1}M^{-1}$)	μ_F (D)	Class
AF488	495	519	2.45	507	71000	7.7	Rhodamine
ATTO488	500	520	2.43	510	90000	8.2	Rhodamine
ATTO495	498	526	2.42	512	80000	9.0	Acridine
DY505	505	530	2.40	517	80000	8.5	Rhodamine
ATTO520	517	538	2.35	527	110000	9.8	Xanthene
AF514	517	542	2.34	529	80000	8.4	Rhodamine
5CR6G	525	557	2.29	541	93000	9.4	Rhodamine
AF532	532	554	2.28	543	81000	8.2	Rhodamine
DY530	539	561	2.25	550	100000	9.2	Rhodamine
TMR	548	574	2.21	561	90000	8.8	Rhodamine
AF546	556	573	2.20	564	112000	9.0	Rhodamine
Cy3B	558	572	2.19	565	130000	11.4	Cyanine
DY560	559	578	2.18	568	120000	9.7	Rhodamine
ATTO565	564	590	2.15	577	120000	10.2	Rhodamine
Cy3.5	578	593	2.12	585	150000	11.9	Cyanine
DY590	580	599	2.10	589	120000	11.5	Rhodamine
AF568	578	603	2.10	590	91300	9.3	Rhodamine
AF594	590	617	2.06	603	90000	9.4	Rhodamine
AF610X	603	623	2.02	613	144000	11.3	Rhodamine
ATTO594	603	626	2.02	614	120000	10.2	Rhodamine
AF633	632	647	1.94	639	100000	9.2	Rhodamine
ATTO633	630	651	1.94	640	130000	10.1	Carborhodamine
ATTO647N	647	664	1.89	655	150000	10.6	Carborhodamine
Cy5	649	665	1.89	657	250000	14.2	Cyanine
AF647	650	665	1.89	657	239000	13.4	Cyanine
DY649P1	655	676	1.86	665	250000	14.0	Cyanine
MR121	661	673	1.86	667	105000	10.2	Oxazine
ATTO655	661	679	1.85	670	125000	11.3	Oxazine
Cy5.5	674	694	1.81	684	250000	14.9	Cyanine
ATTO680	679	698	1.80	688	125000	11.3	Oxazine
AF680	679	702	1.80	690	184000	17.1	Cyanine
DY682	679	708	1.79	693	140000	12.3	Cyanine
AF700	693	714	1.76	703	192000	14.0	Cyanine
ATTO700	700	716	1.75	708	120000	11.1	Oxazine
ATTO725	728	751	1.68	739	120000	11.2	Carborhodamine
DY731	736	760	1.66	748	240000	17.7	Cyanine
ATTO740	743	763	1.65	753	120000	11.9	Carborhodamine
Cy7	750	777	1.62	763	200000	15.0	Cyanine
DY749P1	759	780	1.61	769	240000	16.2	Cyanine
DY778	755	786	1.61	770	240000	17.3	Cyanine
DY800	777	791	1.58	784	280000	16.5	Cyanine
ICG	779	806	1.56	792	262000	17.8	Cyanine

Table S2. Photophysical properties of the investigated fluorophores in water: S_0-S_1 energy gap (ΔE_{00}), fluorescence quantum yield (Φ_{fl}) and excited-state (S_1) lifetime (τ_{S1}) in H_2O and in D_2O, fluorescence enhancement in D_2O, radiative decay rate constant (k_{rad}), solvent-assisted quenching rate constant in H_2O ($k_s = k_{H2O} = \Delta k_{S1}$, measured) as calculated from the experimental data, and solvent quenching rate constant in H_2O as modelled using Förster theory (k_s, calculated). The uncertainty on Φ_{fl} is estimated to ± 5 % and on τ_{S1} to ± 0.05 ns. Uncertainties on the fluorescence enhancement and k_s are obtained by error propagation calculation. The dyes are listed by decreasing ΔE_{00}.

Dye	ΔE_{00} (eV)	$\Phi_{fl}(H_2O)$	$\Phi_{fl}(D_2O)$	$\tau_{S1}(H_2O)$ (ns)	$\tau_{S1}(D_2O)$ (ns)	Fluo. enhancement $\tau_{S1}(D_2O)/\tau_{S1}(H_2O)$	$k_{rad}(H_2O)$ (10^8 s^{-1})	$k_{rad}(D_2O)$ (10^8 s^{-1})	k_s (10^8 s^{-1}) measured	k_{FRET} (10^8 s^{-1}) calculated
AF488	2.45	0.92	0.95	4.02	4.21	1.05 ± 0.02	2.3 ± 0.1	2.3 ± 0.1	0.12 ± 0.06	0.04
ATTO488	2.43	0.80	0.84	4.05	4.26	1.05 ± 0.02	2.0 ± 0.1	2.0 ± 0.1	0.12 ± 0.08	0.05
ATTO495	2.42	0.14	0.15	0.96	1.08	1.12 ± 0.08	1.5 ± 0.1	1.4 ± 0.1	1.1 ± 1.4	0.06
DY505	2.40	0.84	0.92	4.06	4.47	1.10 ± 0.02	2.1 ± 0.1	2.1 ± 0.1	0.22 ± 0.08	0.04
ATTO520	2.35	0.38	0.40	3.69	3.87	1.05 ± 0.02	1.0 ± 0.1	1.0 ± 0.1	0.13 ± 0.10	0.07
AF514	2.34	0.82	0.88	3.98	4.31	1.08 ± 0.02	2.0 ± 0.1	2.0 ± 0.1	0.19 ± 0.08	0.04
5CR6G	2.29	0.85	0.90	3.93	4.23	1.08 ± 0.02	2.2 ± 0.1	2.1 ± 0.1	0.18 ± 0.09	0.05
AF532	2.28	0.46	0.53	2.95	3.29	1.12 ± 0.03	1.5 ± 0.1	1.6 ± 0.1	0.35 ± 0.15	0.06
DY530	2.25	0.83	0.89	3.91	4.29	1.10 ± 0.02	2.1 ± 0.1	2.1 ± 0.1	0.23 ± 0.09	0.09
TMR	2.21	0.30[a]	0.34	1.50	1.73	1.15 ± 0.05	2.0 ± 0.1	2.0 ± 0.1	0.89 ± 0.56	0.07
AF546	2.20	0.85	0.90	3.79	4.18	1.10 ± 0.02	2.2 ± 0.1	2.2 ± 0.1	0.25 ± 0.09	0.09
Cy3B	2.19	0.30	0.33	1.36	1.55	1.14 ± 0.06	2.2 ± 0.1	2.1 ± 0.1	0.90 ± 0.68	0.03
DY560	2.18	0.27	0.30	1.67	1.80	1.08 ± 0.04	1.6 ± 0.1	1.6 ± 0.1	0.46 ± 0.47	0.16
ATTO565	2.15	0.56	0.59	3.90	4.35	1.11 ± 0.02	1.4 ± 0.1	1.3 ± 0.1	0.26 ± 0.08	0.17
Cy3.5	2.12	0.10	0.12	0.51	0.58	1.14 ± 0.15	1.9 ± 0.2	2.0 ± 0.2	2.4 ± 4.9	0.09
DY590	2.10	0.76	0.91	3.01	3.82	1.27 ± 0.03	2.5 ± 0.1	2.4 ± 0.1	0.70 ± 0.13	0.30
AF568	2.10	0.56	0.70	3.43	4.00	1.16 ± 0.02	1.6 ± 0.1	1.8 ± 0.1	0.41 ± 0.11	0.14
AF594	2.06	0.59	0.69	3.84	4.37	1.14 ± 0.02	1.5 ± 0.1	1.6 ± 0.1	0.31 ± 0.09	0.18
AF610X	2.02	0.84	0.96	3.66	4.34	1.18 ± 0.02	2.3 ± 0.1	2.2 ± 0.1	0.42 ± 0.09	0.35
ATTO594	2.02	0.81	0.93	3.89	4.54	1.17 ± 0.02	2.1 ± 0.1	2.1 ± 0.1	0.37 ± 0.08	0.32
AF633	1.94	0.65	0.78	3.24	4.09	1.26 ± 0.02	2.0 ± 0.1	1.9 ± 0.1	0.64 ± 0.11	0.52
ATTO633	1.94	0.55	0.73	3.31	4.64	1.40 ± 0.03	1.6 ± 0.1	1.6 ± 0.1	0.87 ± 0.10	0.74
ATTO647N	1.89	0.64	0.84	3.71	4.77	1.28 ± 0.02	1.7 ± 0.1	1.8 ± 0.1	0.59 ± 0.08	1.0
Cy5	1.89	0.29	0.35	0.99	1.19	1.20 ± 0.08	3.0 ± 0.2	2.9 ± 0.2	1.7 ± 1.2	1.1
AF647	1.89	0.34	0.41	1.02	1.21	1.19 ± 0.08	3.3 ± 0.2	3.4 ± 0.2	1.5 ± 1.2	1.0
DY649P1	1.86	0.37	0.50	1.09	1.36	1.25 ± 0.07	3.4 ± 0.2	3.7 ± 0.2	1.8 ± 1.0	1.9
MR121	1.86	0.17	0.27	1.89	3.85	2.04 ± 0.06	0.9 ± 0.1	0.7 ± 0.1	2.7 ± 0.3	1.4
ATTO655	1.85	0.28	0.56	1.94	3.92	2.02 ± 0.06	1.4 ± 0.1	1.4 ± 0.1	2.6 ± 0.3	2.3
Cy5.5	1.81	0.24	0.36	0.86	1.22	1.42 ± 0.10	2.8 ± 0.2	2.9 ± 0.2	3.4 ± 1.5	3.2
ATTO680	1.80	0.30	0.62	1.86	4.00	2.15 ± 0.06	1.6 ± 0.1	1.5 ± 0.1	2.9 ± 0.3	3.4
AF680	1.80	0.36	0.57	1.17	1.71	1.46 ± 0.08	3.1 ± 0.2	3.3 ± 0.2	2.7 ± 0.8	4.9
DY682	1.79	0.20	0.44	1.10	2.35	2.14 ± 0.11	1.8 ± 0.1	1.9 ± 0.1	4.8 ± 0.8	3.9
AF700	1.76	0.26	0.46	0.93	1.61	1.72 ± 0.11	2.7 ± 0.2	2.9 ± 0.2	4.5 ± 1.2	5.8
ATTO700	1.75	0.25	0.62	1.74	4.06	2.34 ± 0.07	1.5 ± 0.1	1.5 ± 0.1	3.3 ± 0.3	5.3
ATTO725	1.68	0.05	0.13	0.49	1.29	2.63 ± 0.29	1.1 ± 0.1	1.0 ± 0.1	13 ± 4	8.2
DY731	1.66	0.09	0.17	0.46	0.76	1.66 ± 0.21	2.0 ± 0.2	2.2 ± 0.2	8.7 ± 5.1	20
ATTO740	1.65	0.07	0.18	0.66	1.67	2.52 ± 0.20	1.1 ± 0.1	1.1 ± 0.1	9.1 ± 2.3	9.8
Cy7	1.62	0.12	0.32	0.55	1.31	2.38 ± 0.23	2.1 ± 0.2	2.4 ± 0.2	11 ± 3	15
DY749P1	1.61	0.12	0.32	0.55	1.34	2.45 ± 0.24	2.2 ± 0.2	2.4 ± 0.1	11 ± 3	17
DY778	1.61	0.02	0.04	<IRF	0.33	ND	ND	ND	ND	ND
DY800	1.58	0.08	0.17	0.37	0.87	2.38 ± 0.35	2.1 ± 0.3	2.0 ± 0.2	16 ± 8	17
ICG	1.56	0.02[b]	0.07	0.20	0.62	3.05 ± 0.80	0.9 ± 0.2	1.1 ± 0.1	33 ± 25	21

[a]From ref. S21. [b]Determined against ICG in DMSO (Φ_{fl} = 0.11).[S22] ND: not determined.

Table S3. Photophysical properties of various fluorophores in methanol: S_0-S_1 energy gap in water (ΔE_{00}), fluorescence quantum yield (Φ_{fl}) and excited-state (S_1) lifetime (τ_{S1}) in MeOH and in MeOH-d4, fluorescence enhancement in MeOH-d4, radiative decay rate constant (k_{rad}), solvent-assisted quenching rate constant in MeOH ($k_s = k_{MeOH} = \Delta k_{S1}$) as calculated from the experimental data, and solvent quenching rate constant in MeOH as modelled using Förster theory (k_s, calculated). The uncertainty on Φ_{fl} is estimated to ± 5 % and on τ_{S1} to ± 0.05 ns. Uncertainties on the fluorescence enhancement and k_s are obtained by error propagation. The dyes are listed by decreasing ΔE_{00}.

Dye	ΔE_{00} (eV)	Φ_{fl} (MeOH)	Φ_{fl} (MeOH-d4)	τ_{S1} (MeOH) (ns)	τ_{S1} (MeOH-d4) (ns)	Fluo. enhancement τ_{S1}(MeOH-d4)/ τ_{S1}(MeOH)	k_{rad} (MeOH) (10^8 s^{-1})	k_{rad} (MeOH-d4) (10^8 s^{-1})	k_s (10^8 s^{-1}) measured	k_{FRET} (10^8 s^{-1}) calculated
AF488	2.45	0.81	0.91	4.20	4.40	1.05 ± 0.02	1.9 ± 0.1	2.1 ± 0.1	0.19 ± 0.08	0.01
DY505	2.40	0.96	1.00	4.24	4.39	1.04 ± 0.02	2.3 ± 0.1	2.3 ± 0.1	0.08 ± 0.08	0.01
ATTO520	2.35	0.45	0.47	3.85	4.03	1.05 ± 0.02	1.2 ± 0.1	1.2 ± 0.1	0.12 ± 0.09	0.02
AF532	2.28	0.40	0.52	2.59	2.93	1.13 ± 0.03	1.5 ± 0.1	1.8 ± 0.1	0.45 ± 0.19	0.02
DY530	2.25	0.91	0.97	4.13	4.34	1.05 ± 0.02	2.2 ± 0.1	2.2 ± 0.1	0.12 ± 0.08	0.03
TMR	2.21	0.65	0.66	2.72	2.74	1.01 ± 0.03	2.4 ± 0.1	2.4 ± 0.1	0.04 ± 0.19	0.02
DY560	2.18	0.43	0.47	2.40	2.44	1.02 ± 0.03	1.8 ± 0.1	1.9 ± 0.1	0.08 ± 0.24	0.04
Cy3.5	2.12	0.15	0.15	0.74	0.77	1.04 ± 0.10	2.1 ± 0.2	2.0 ± 0.2	0.6 ± 2.5	0.02
AF568	2.10	0.62	0.71	3.85	4.26	1.11 ± 0.02	1.6 ± 0.1	1.7 ± 0.1	0.25 ± 0.09	0.04
ATTO594	2.02	0.94	1.06	4.44	4.80	1.08 ± 0.02	2.1 ± 0.1	2.2 ± 0.1	0.17 ± 0.07	0.09
ATTO633	1.94	0.70	0.85	4.31	5.01	1.16 ± 0.02	1.6 ± 0.1	1.7 ± 0.1	0.32 ± 0.07	0.20
ATTO647N	1.89	0.85	0.95	4.39	4.85	1.10 ± 0.02	1.9 ± 0.1	2.0 ± 0.1	0.21 ± 0.07	0.29
AF647	1.89	0.83	0.92	1.47	1.58	1.07 ± 0.05	5.7 ± 0.3	5.8 ± 0.3	0.47 ± 0.61	0.28
DY649P1	1.86	0.64	0.74	1.85	2.09	1.13 ± 0.04	3.5 ± 0.2	3.5 ± 0.2	0.63 ± 0.37	0.52
ATTO655	1.85	0.56	0.76	3.43	4.54	1.32 ± 0.02	1.6 ± 0.1	1.7 ± 0.1	0.71 ± 0.10	0.64
Cy5.5	1.81	0.32	0.41	1.30	1.61	1.24 ± 0.06	2.4 ± 0.2	2.5 ± 0.1	1.5 ± 0.7	0.89
ATTO680	1.80	0.54	0.88	3.17	4.55	1.44 ± 0.03	1.7 ± 0.1	1.9 ± 0.1	0.96 ± 0.11	0.95
ATTO700	1.75	0.48	0.85	3.05	4.59	1.50 ± 0.03	1.6 ± 0.1	1.8 ± 0.1	1.1 ± 0.1	1.5
ATTO725	1.68	0.14	0.23	1.15	1.89	1.65 ± 0.08	1.2 ± 0.1	1.2 ± 0.1	3.4 ± 0.8	2.3
ATTO740	1.65	0.18	0.30	1.42	2.23	1.57 ± 0.07	1.3 ± 0.1	1.3 ± 0.1	2.5 ± 0.5	2.7
DY749P1	1.61	0.21	0.36	1.08	1.76	1.63 ± 0.09	2.0 ± 0.1	2.0 ± 0.1	3.6 ± 0.9	4.8
DY778	1.61	0.07	0.12	0.44	0.59	1.34 ± 0.19	1.5 ± 0.2	2.0 ± 0.2	5.8 ± 5.9	5.3
DY800	1.58	0.24	0.34	1.04	1.56	1.50 ± 0.09	2.3 ± 0.2	2.2 ± 0.1	3.2 ± 1.0	4.7
ICG	1.56	0.06	0.12	0.61	1.09	1.77 ± 0.17	1.0 ± 0.1	1.1 ± 0.1	7.1 ± 2.8	5.9

Table S4. Photophysical properties of the fluorophore ATTO655 in various solvents: solvent refractive index (n), Kamlet-Taft solvent acidity parameter α,[S23] fluorescence quantum yield (Φ_{fl}), excited-state lifetime (τ_{S1}), radiative decay rate constant (k_{rad}), calculated solvent-assisted quenching rate constant (k_s) using for k_{nr} the value determined in acetonitrile-d3 ($6.8 \cdot 10^7$ s^{-1}). The uncertainty on the lifetimes is estimated to ± 0.05 ns. The uncertainties on τ_{rad} and k_s was obtained by error propagation. ND: not determined.

Solvent	n	α	Φ_{fl}	τ_{S1} (ns)	k_{rad} (10^8 s^{-1})	k_s (10^8 s^{-1})
H_2O	1.332	1.17	0.28	1.94	1.4 ± 0.1	3.0 ± 0.2
D_2O	1.328		0.56	3.92	1.4 ± 0.1	0.44 ± 0.11
Ethyleneglycol	1.4318	0.90	0.42	2.93	1.4 ± 0.1	1.3 ± 0.1
CH_3OH	1.3288	0.98	0.52	3.34	1.6 ± 0.1	0.74 ± 0.12
CD_3OH	1.327		0.56	3.69	1.5 ± 0.1	0.52 ± 0.12
CH_3OD	1.327		0.65	4.11	1.6 ± 0.1	0.18 ± 0.12
CD_3OD	1.326		0.72	4.52	1.6 ± 0.1	0.00 ± 0.11
Ethanol	1.3611	0.86	0.58	3.62	1.6 ± 0.1	0.48 ± 0.12
Ethanol-d1	1.359		0.67	4.16	1.6 ± 0.1	0.10 ± 0.12
Ethanol-d6	1.358		0.73	4.52	1.6 ± 0.1	0.00 ± 0.12
1-propanol	1.385	0.84	0.57	3.64	1.6 ± 0.1	0.50 ± 0.12
2-propanol	1.3776	0.76	0.63	3.78	1.7 ± 0.1	0.30 ± 0.12
HFIP	1.275	1.96	0.45	3.66	1.2 ± 0.1	0.81 ± 0.11
1-butanol	1.3988	0.84	0.55	3.72	1.5 ± 0.1	0.52 ± 0.11
1-pentanol	1.4101	0.84	0.59	3.75	1.6 ± 0.1	0.40 ± 0.12
1-hexanol	1.4178	0.80	0.60	3.88	1.6 ± 0.1	0.34 ± 0.12
1-heptanol	1.4249		0.59	3.82	1.5 ± 0.1	0.39 ± 0.12
Acetone	1.3588	0.08	0.62	4.40	1.4 ± 0.1	0.17 ± 0.11
Acetone-d6	1.355		ND	4.55	ND	ND
Acetonitrile	1.3442	0.19	0.67	4.50	1.5 ± 0.1	0.04 ± 0.11
Acetonitrile-d3	1.344		0.68	4.74	1.4 ± 0.1	0
Nitromethane	1.3817	0.22	0.66	4.34	1.5 ± 0.1	0.11 ± 0.11
Nitromethane-d3	1.3935		0.67	4.56	1.5 ± 0.1	0.04 ± 0.11

Table S5. Excited-state (S_1) lifetime (τ_{S1}) of various fluorophores in 2-propanol (*i*PrOH) and in hexafluoroisopropanol (HFIP). The uncertainty on the lifetimes is estimated to ± 0.05 ns.

Dye	ΔE_{00} (eV)	$\tau_{S1}(H_2O)$ (ns)	τ_{S1}(*i*PrOH) (ns)	τ_{S1}(HFIP) (ns)
AF488	2.45	4.02	3.71	4.36
AF568	2.10	3.43	3.62	3.97
ATTO647N	1.89	3.71	4.28	4.98
ATTO655	1.85	1.94	3.78	3.66
ATTO700	1.75	1.74	3.58	3.46
ATTO740	1.65	0.66	1.73	1.80
DY749P1	1.61	0.55	1.36	1.69
ICG	1.56	0.20	0.82	0.81

Table S6. List of vibrational frequencies (expressed in wavenumbers) of fundamental and combination modes for H_2O as obtained from decomposing the H_2O absorption spectrum with a sum of Gaussian components ($\bar{\nu}_{fit}$) and tentative assignment obtained by combining the fundamental frequencies, or as found in the literature ($\bar{\nu}$)[S24, 25].

$\bar{\nu}_{fit}$ (cm^{-1})	$\bar{\nu}$ (cm^{-1})	Assignment
	1645^{a}	ν_2 (bending)
	3277^{a}	ν_1 (symmetric stretching)
	3490^{a}	ν_3 (asymmetric stretching)
10260	10300^{a}	a ν_1 + b ν_3; a + b = 3
11150	11470^{b}	3 ν_1 + ν_2
11760	11690-12115^{b}	a ν_1 + ν_2 + b ν_3; a + b = 3, b ≥ 1
13050	13160^{a}	3 ν_1 + ν_3
13530	13510^{a}	ν_1 + 3 ν_3
15150	15150^{a}	a ν_1 + ν_2 + b ν_3; a + b = 4

^{a}Ref. S24. ^{b}Tentative assignment

Table S7. Energy transfer related photophysical quantities of the investigated fluorophores: S_0-S_1 energy gap (ΔE_{00}), Förster radius (R_0) calculated in water, and FRET efficiency (Φ_{FRET}) in H_2O and in MeOH. The uncertainty on Φ_{FRET} was obtained by error propagation. The dyes are listed by decreasing ΔE_{00}. ND: not determined.

Dye	ΔE_{00} (eV)	$R_0(H_2O)$ (nm)	$\Phi_{FRET}(H_2O)$	Φ_{FRET}(MeOH)
AF488	2.45	0.18	0.05 ± 0.03	0.010 ± 0.007
ATTO488	2.43	0.17	0.05 ± 0.03	ND
ATTO495	2.42	0.13	0.11 ± 0.13	ND
DY505	2.40	0.17	0.09 ± 0.03	0.008 ± 0.008
ATTO520	2.35	0.15	0.05 ± 0.04	0.005 ± 0.004
AF514	2.34	0.17	0.08 ± 0.03	ND
5CR6G	2.29	0.17	0.07 ± 0.03	ND
AF532	2.28	0.17	0.10 ± 0.04	0.02 ± 0.01
DY530	2.25	0.19	0.09 ± 0.03	0.011 ± 0.008
TMR	2.21	0.16	0.13 ± 0.08	0.003 ± 0.013
AF546	2.20	0.19	0.09 ± 0.03	ND
Cy3B	2.19	0.13	0.12 ± 0.09	ND
DY560	2.18	0.17	0.08 ± 0.08	0.004 ± 0.011
ATTO565	2.15	0.19	0.10 ± 0.03	ND
Cy3.5	2.12	0.13	0.12 ± 0.25	0.01 ± 0.04
DY590	2.10	0.17	0.21 ± 0.04	ND
AF568	2.10	0.19	0.14 ± 0.04	0.018 ± 0.006
AF594	2.06	0.13	0.12 ± 0.03	ND
AF610X	2.02	0.21	0.16 ± 0.03	ND
ATTO594	2.02	0.19	0.14 ± 0.03	0.017 ± 0.007
AF633	1.94	0.20	0.21 ± 0.04	ND
ATTO633	1.94	0.22	0.29 ± 0.03	0.027 ± 0.006
ATTO647N	1.89	0.22	0.22 ± 0.03	0.020 ± 0.006
Cy5	1.89	0.23	0.17 ± 0.12	ND
AF647	1.89	0.23	0.16 ± 0.12	0.04 ± 0.05
DY649P1	1.86	0.24	0.20 ± 0.11	0.04 ± 0.03
MR121	1.86	0.20	0.51 ± 0.06	ND
ATTO655	1.85	0.20	0.50 ± 0.05	0.05 ± 0.01
Cy5.5	1.81	0.22	0.30 ± 0.13	0.06 ± 0.03
ATTO680	1.80	0.27	0.54 ± 0.06	0.08 ± 0.01
AF680	1.80	0.24	0.32 ± 0.10	ND
DY682	1.79	0.25	0.53 ± 0.10	ND
AF700	1.76	0.25	0.42 ± 0.12	ND
ATTO700	1.75	0.28	0.57 ± 0.06	0.08 ± 0.01
ATTO725	1.68	0.23	0.62 ± 0.22	0.07 ± 0.02
DY731	1.66	0.24	0.40 ± 0.24	ND
ATTO740	1.65	0.25	0.60 ± 0.16	0.07 ± 0.01
Cy7	1.62	0.27	0.58 ± 0.19	ND
DY749P1	1.61	0.27	0.59 ± 0.19	0.11 ± 0.03
DY778	1.61	0.19	ND	0.06 ± 0.06
DY800	1.58	0.25	0.58 ± 0.29	0.10 ± 0.03
ICG	1.56	0.22	0.67 ± 0.53	0.08 ± 0.03

7. Supplementary Figures

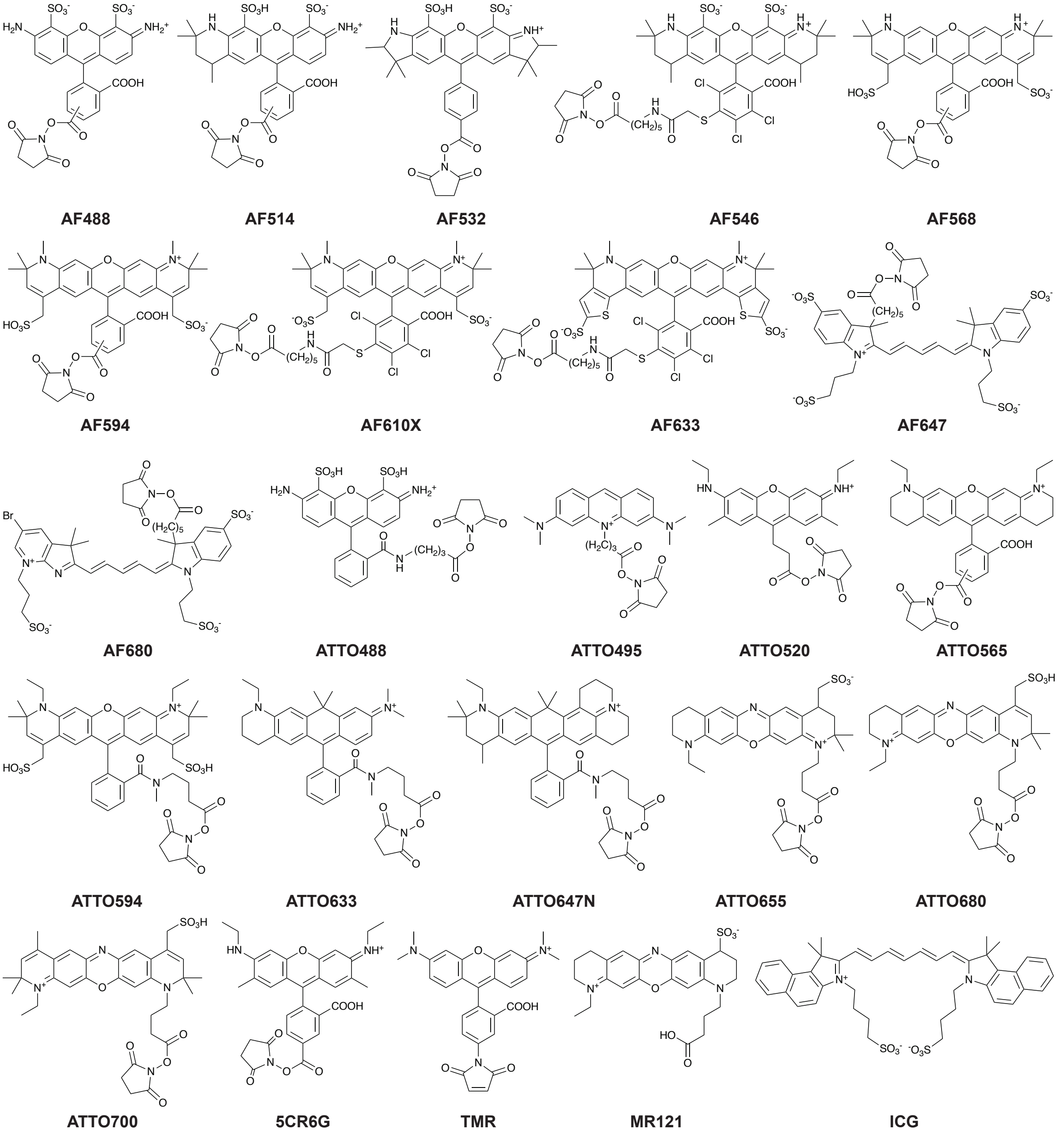

Figure S1. Molecular structures of the investigated Alexa Fluor (AF) and ATTO dyes shown as NHS-ester derivatives, as well as of 5-carboxyrhodamine 6G (5CR6G) NHS ester, tetramethylrhodamine (TMR) maleimide, MR121, and indocyanine green (ICG). The structures of AF700, ATTO725, and ATTO740 are proprietary and therefore unavailable.

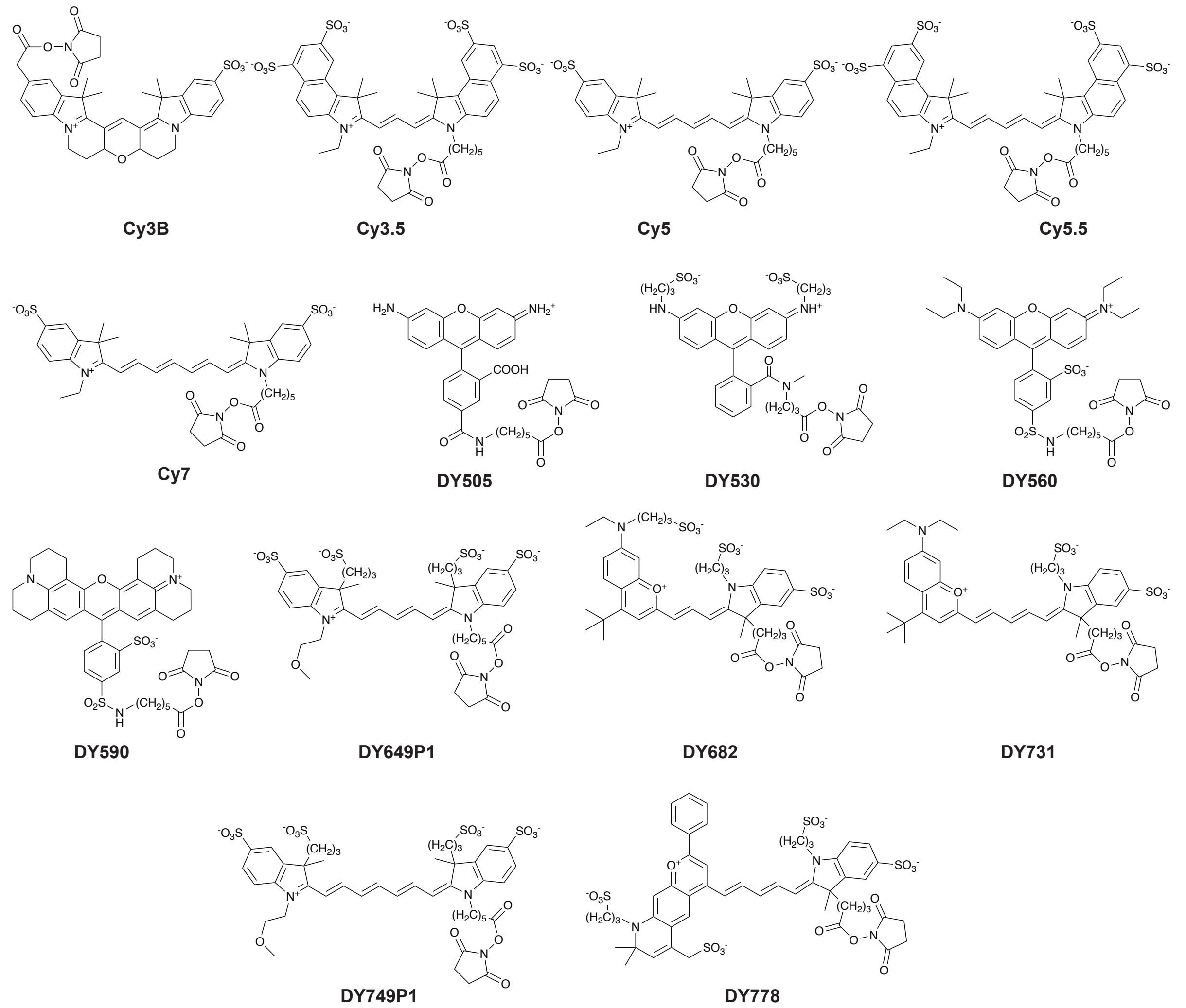

Figure S2. Molecular structures of the investigated Cy and Dyomics (DY) dyes, shown as NHS-ester derivatives. The structure of DY800 is proprietary and therefore unavailable.

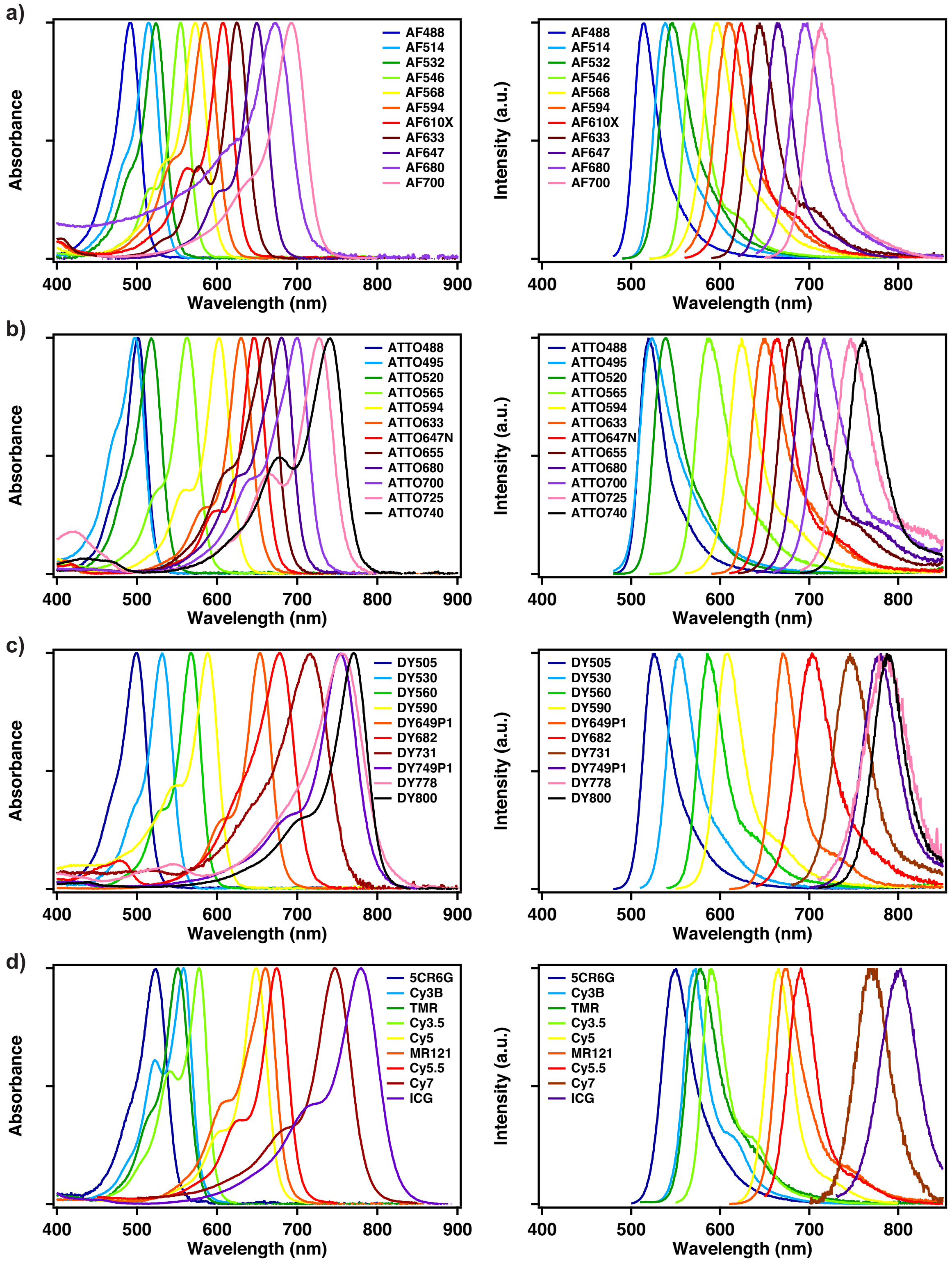

Figure S3. Intensity-normalized absorption (left) and fluorescence (right) spectra of the investigated dyes in H_2O. (a) Alexa Fluor (AF) dyes. (b) ATTO dyes. (c) Dyomics dyes. (d) Other dyes. Abbreviations: 5CR6G: 5-carboxyrhodamine 6G; TMR: tetramethylrhodamine; ICG: indocyanine green.

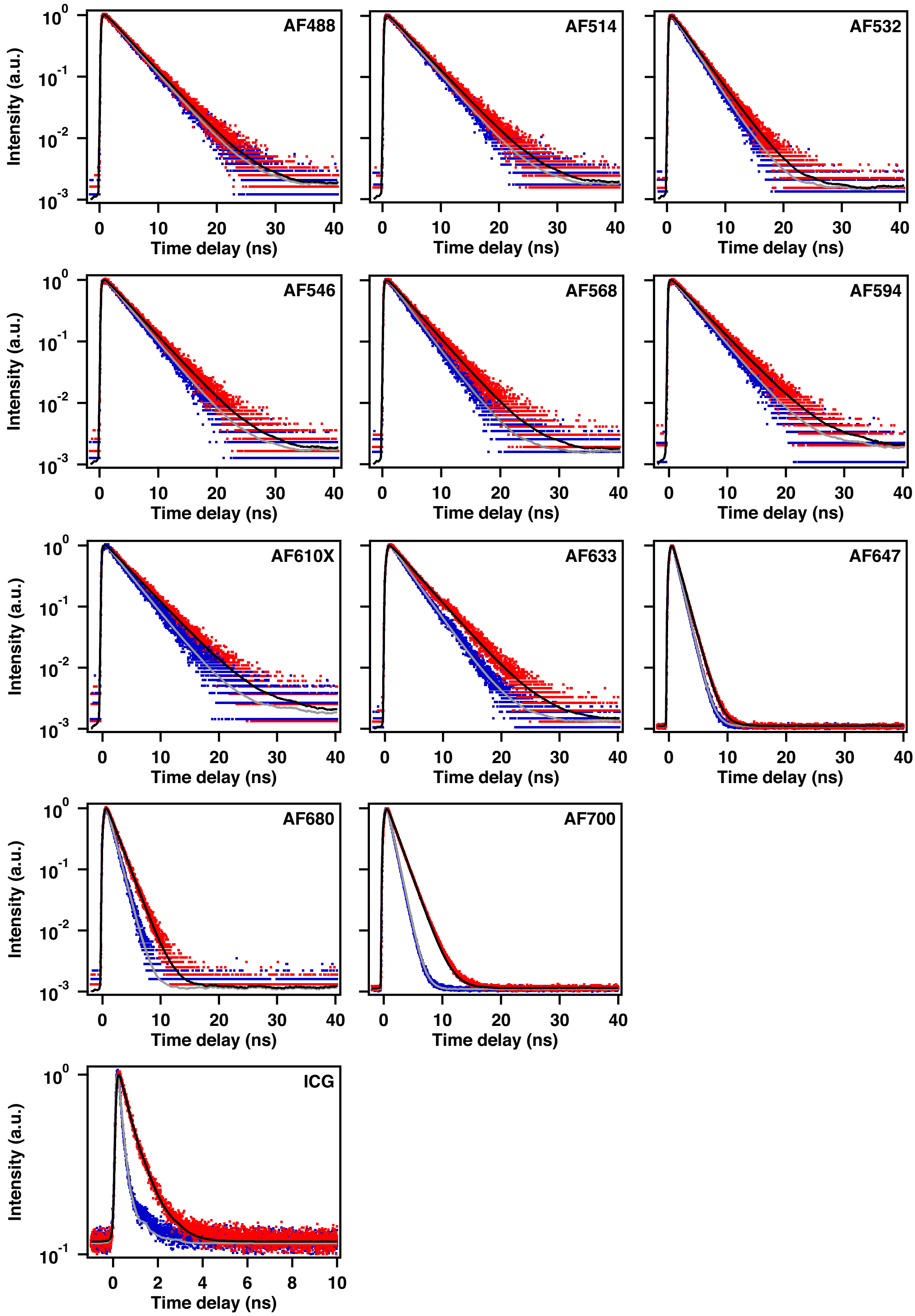

Figure S4. Intensity-normalized fluorescence time profile of Alexa Fluor fluorophores and of ICG in H_2O (blue) and in D_2O (red). Solid lines are exponential fits to the data points (grey: H_2O; black: D_2O).

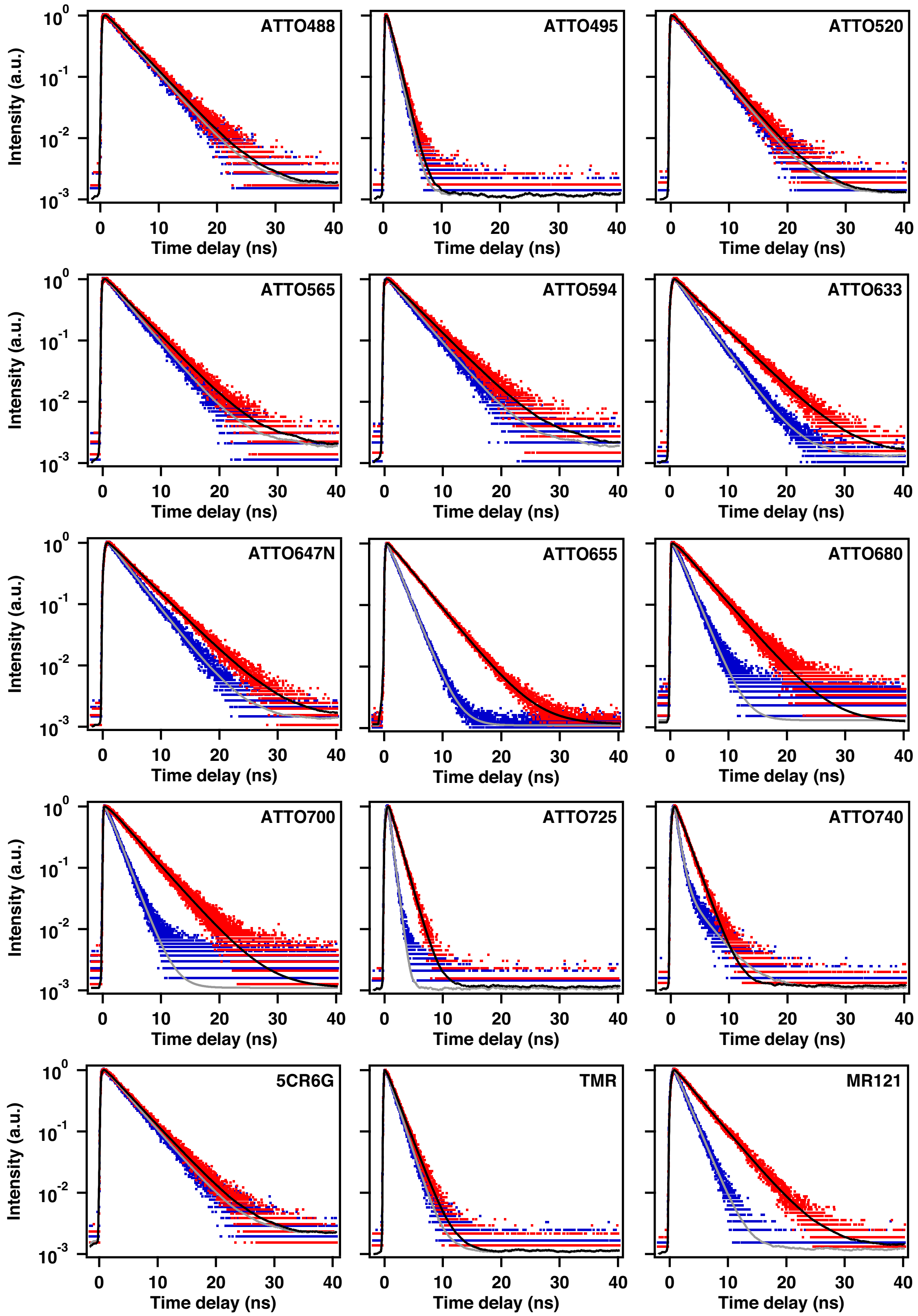

Figure S5. Intensity-normalized fluorescence time profile of ATTO and of some other fluorophores in H_2O (blue) and in D_2O (red). Solid lines are exponential fits to the data points (grey: H_2O; black: D_2O).

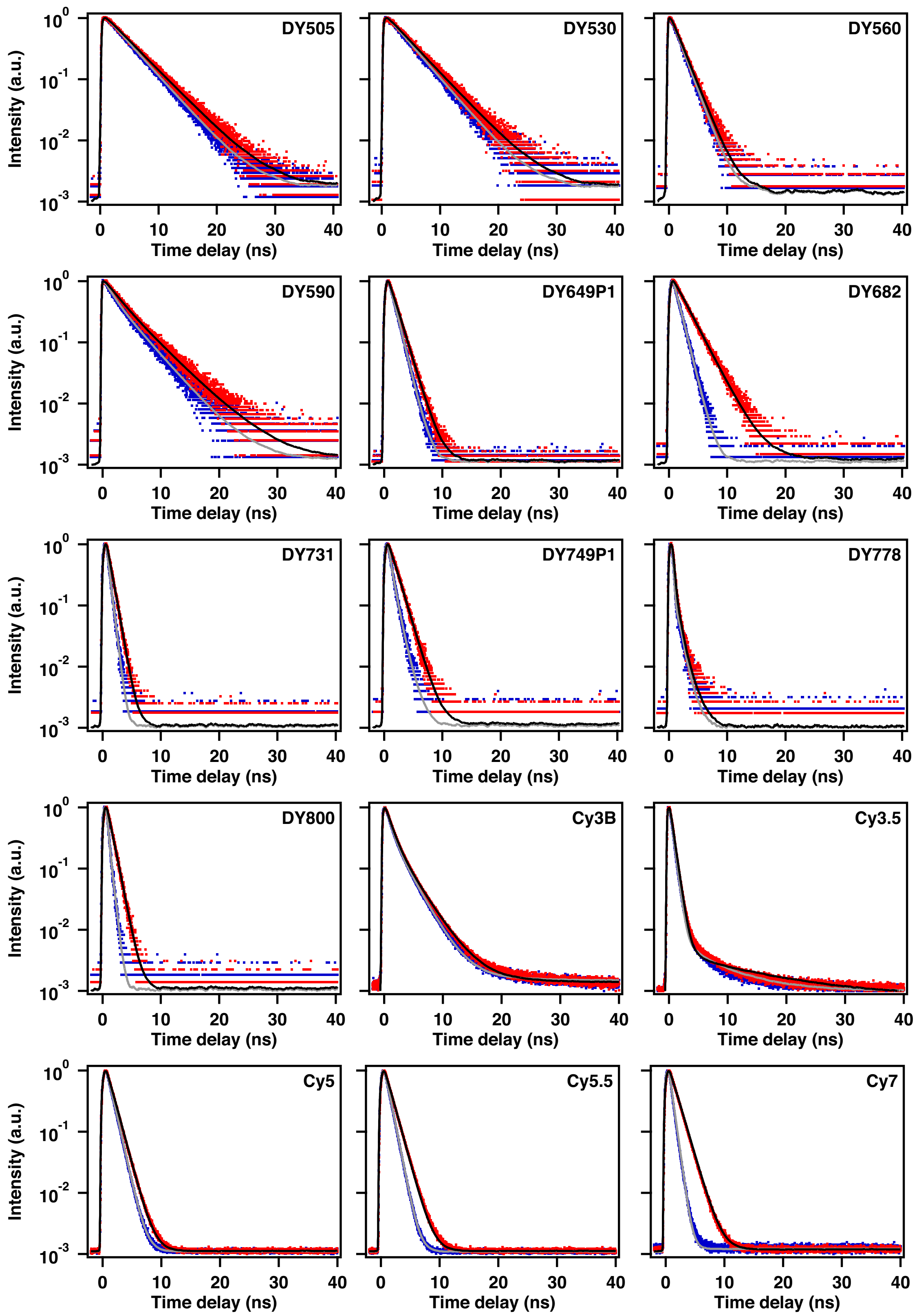

Figure S6. Intensity-normalized fluorescence time profile of DY and Cy fluorophores in H_2O (blue) and in D_2O (red). Solid lines are exponential fits to the data points (grey: H_2O; black: D_2O).

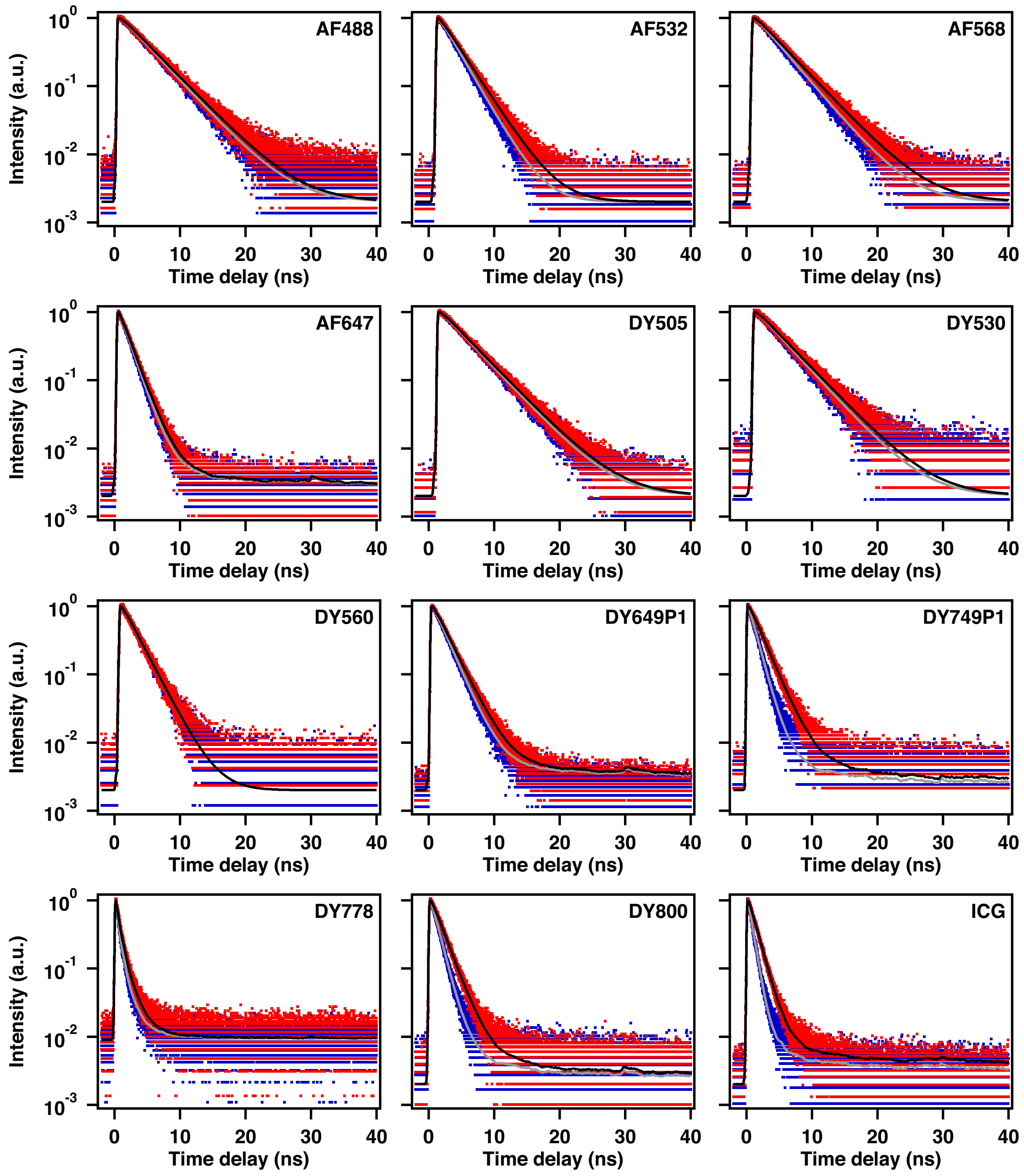

Figure S7. Intensity-normalized fluorescence time profile of Alexa Fluor and DY fluorophores as well as of ICG in methanol (blue) and in methanol-d4 (red). Solid lines are exponential fits to the data points (grey: methanol; black: methanol-d4).

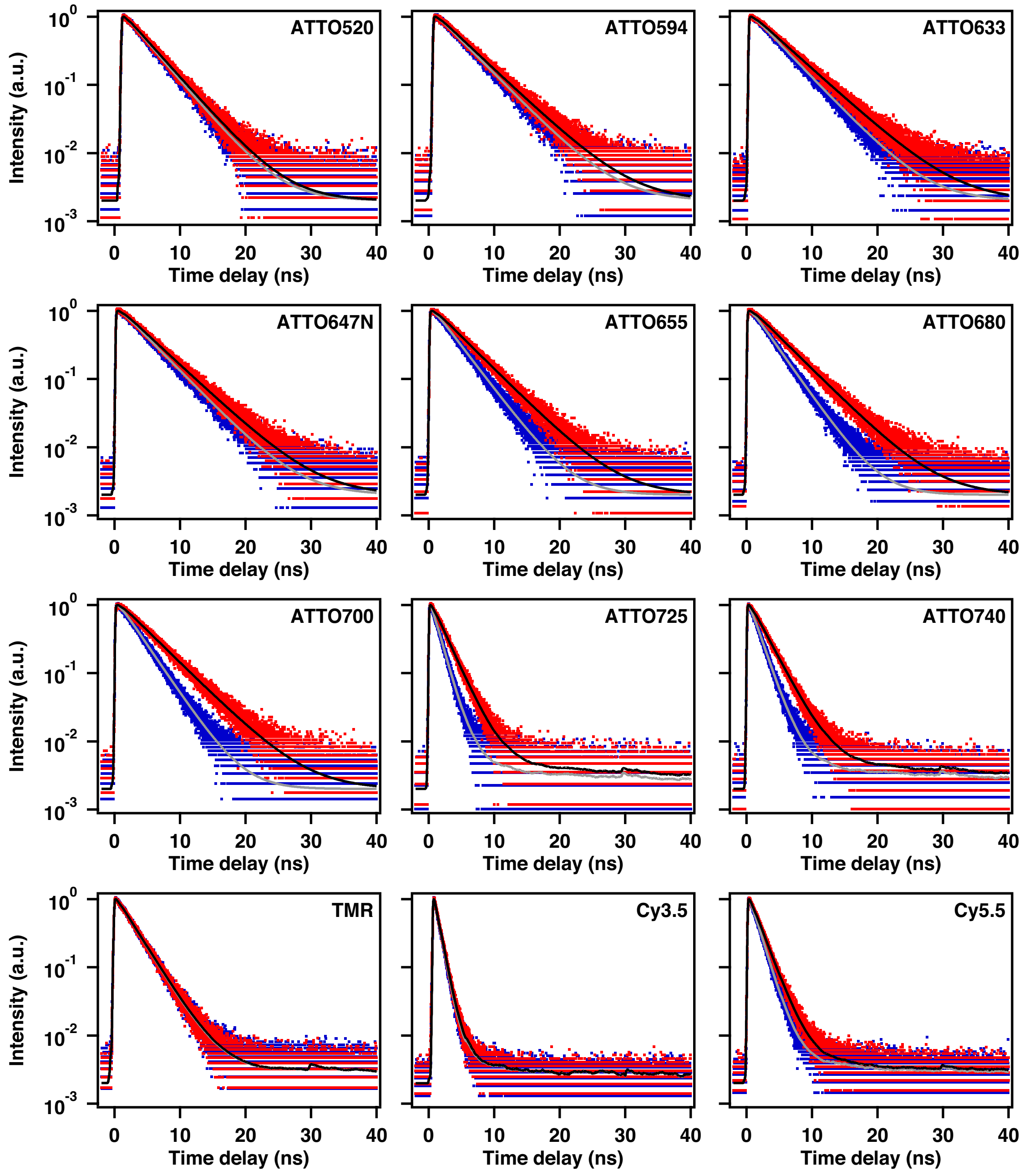

Figure S8. Intensity-normalized fluorescence time profile of ATTO and Cy fluorophores as well as of TMR in methanol (blue) and in methanol-d4 (red). Solid lines are exponential fits to the data points (grey: methanol; black: methanol-d4).

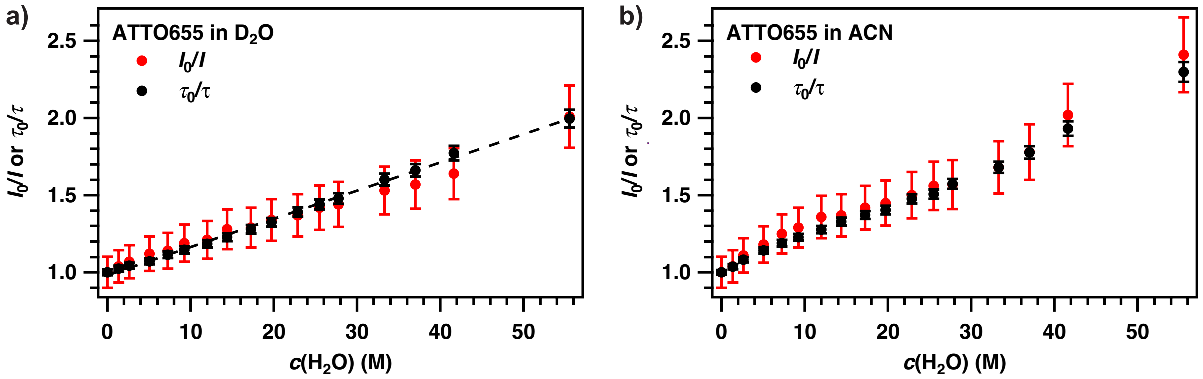

Figure S9. Stern-Volmer plots of the quenching of ATTO655 by H_2O in D_2O (a) and in ACN (b). Red circles represent fluorescence intensity variations and black circles excited-state lifetime variations upon increasing concentrations of H_2O. Error bars were obtained from the estimated uncertainty on intensity and lifetime measurements. The dashed line in (a) is a linear fit to the lifetime data using the Stern-Volmer equation. The fact that the measured points in (b) do not follow a straight line is an indication of preferential solvation of the dye by H_2O rather than by ACN.

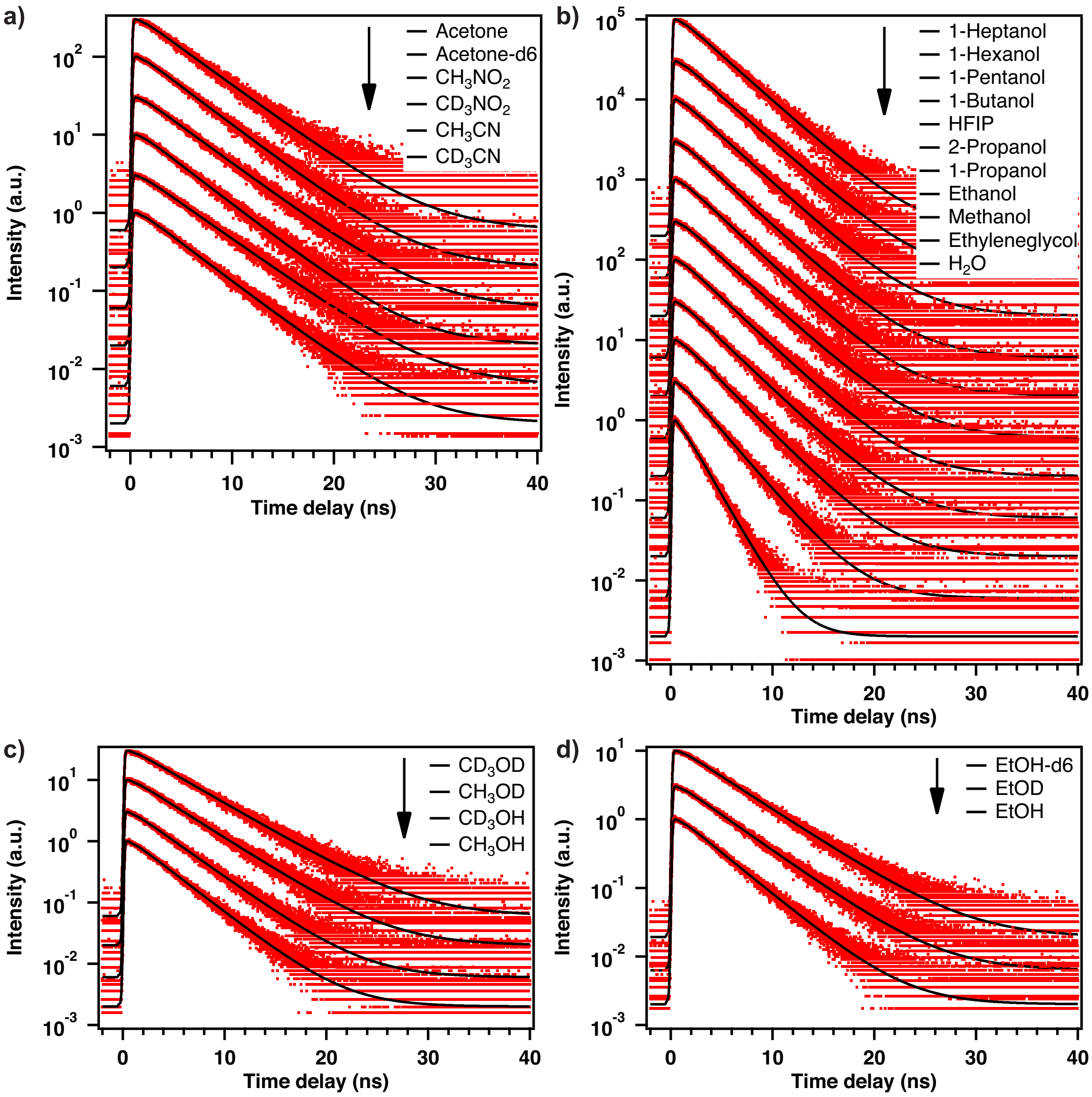

Figure S10. Intensity-normalized fluorescence time profile of ATTO655 in (a) non-alcoholic solvents, (b) water and alcohols, (c) methanol, and (d) ethanol. Black lines represent best exponential fits to the data points. Curves are shifted on the vertical axis for the sake of clarity.

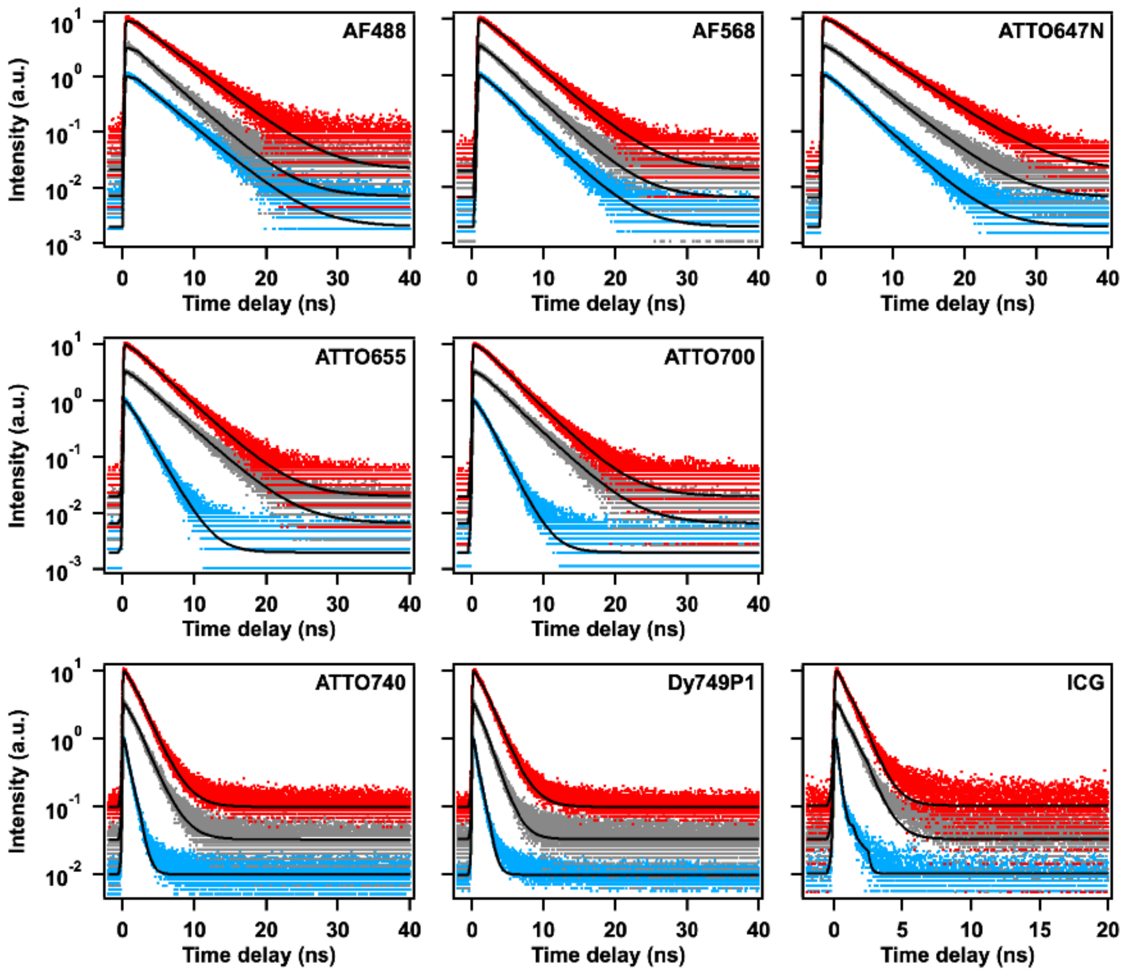

Figure S11. Intensity-normalized fluorescence time profile of various fluorophores in H_2O (blue), in 2-propanol (grey), and in HFIP (red). Black lines represent best exponential fits to the data points. Curves are shifted on the vertical axis for the sake of clarity.

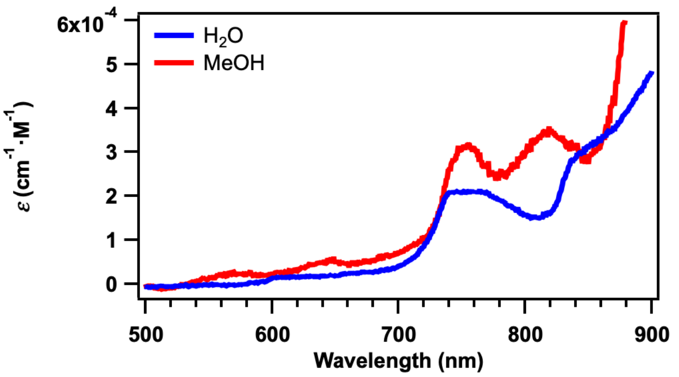

Figure S12. Absorption spectrum of pure, liquid H_2O and MeOH.

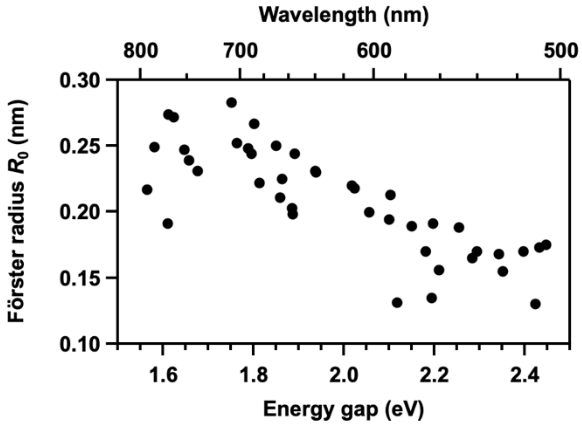

Figure S13. Calculated Förster radius (R_0) for all investigated fluorophores in water.

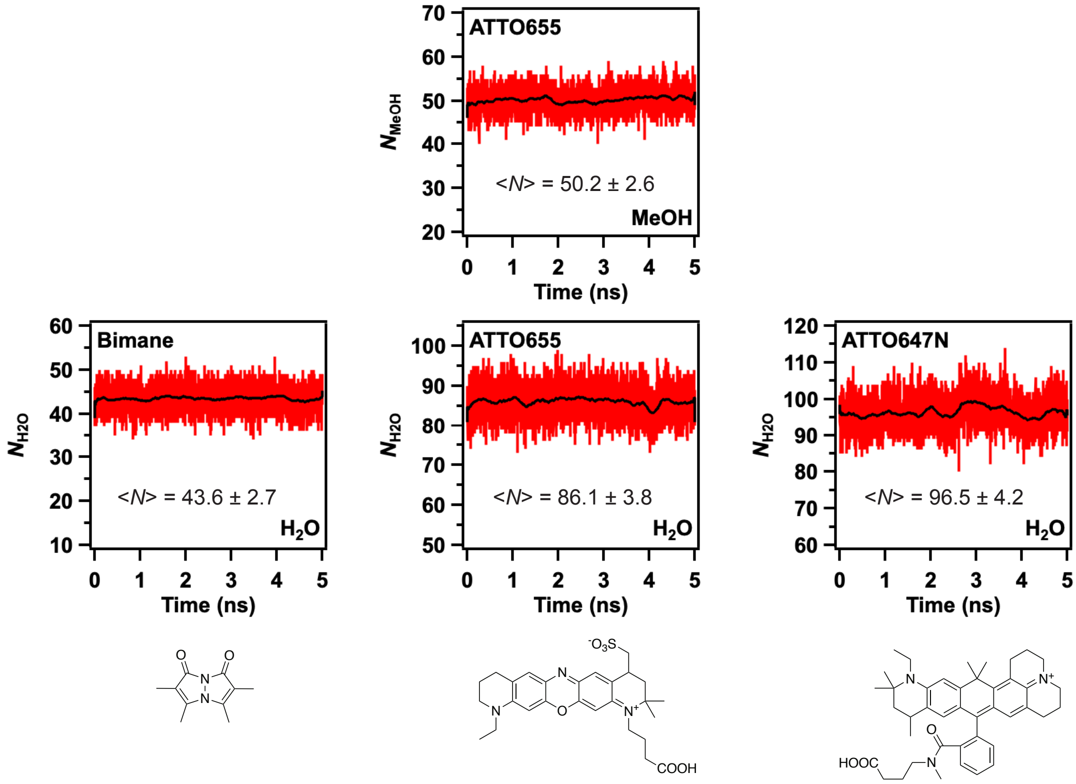

Figure S14. Number of methanol molecules (N_{MeOH}) and number of water molecules (N_{H2O}) inside the first solvent shell of the fluorophores bimane, ATTO655, and ATTO647N as a function of time obtained from MD simulations, and their average number over the total time window (<*N*>). The uncertainties reported are standard deviations. Black traces were obtained by smoothing red data points. The molecular structures of the fluorophores are drawn to highlight differences in size.

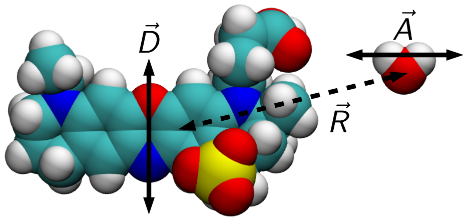

Figure S15. Vectors used to define the relative orientation of water and fluorophore molecules in order to calculate the orientation factor κ^2.

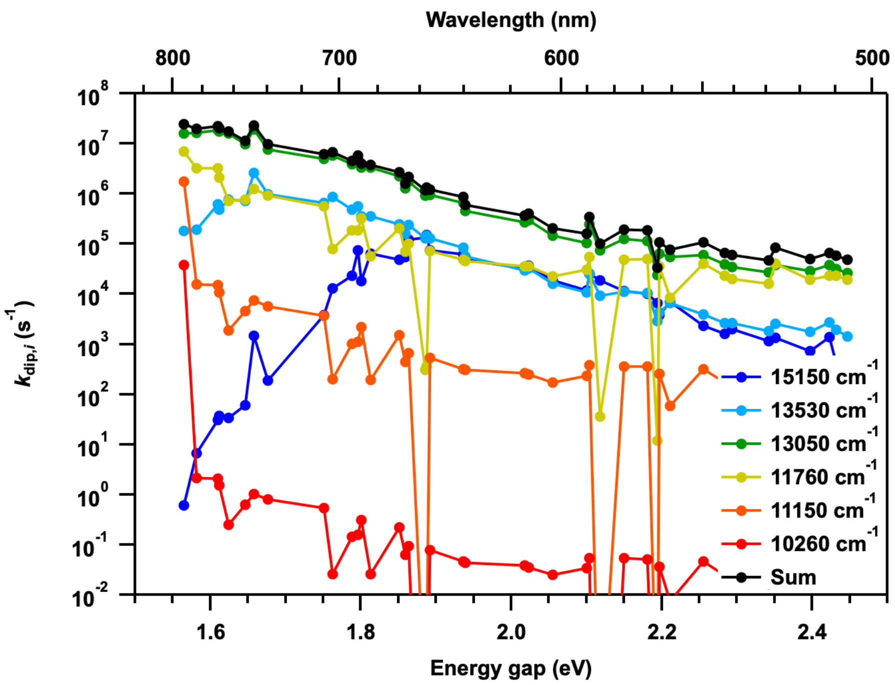

Figure S16. Calculated energy transfer decay rate constant k_{dip} between every component (i) of the water spectrum (characterized by its central frequency) and every dye (represented by a full circle at its energy gap value). The sum of all components (black line) represents k_{FRET} shown in Figure 5f.

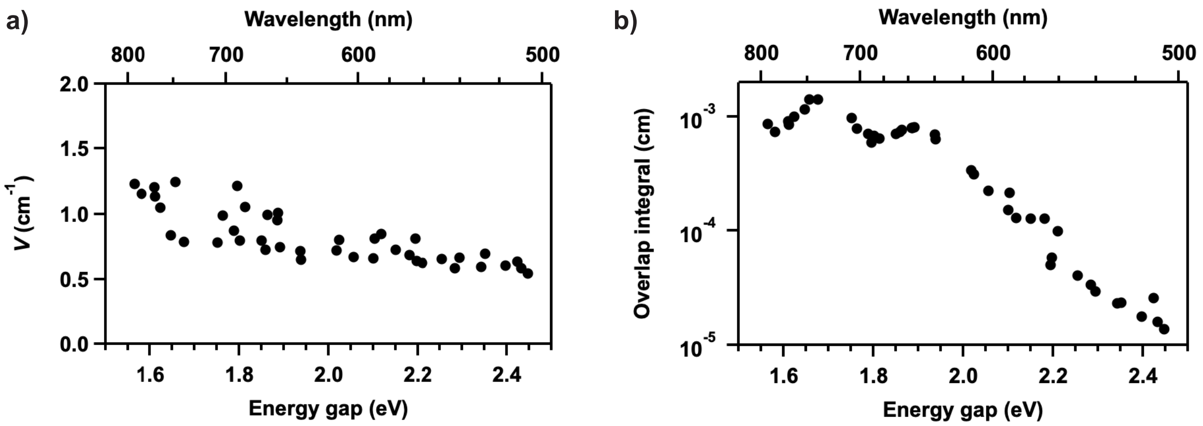

Figure S17. (a) Total dipole-dipole coupling energy V between fluorophores and water calculated using eq. 6 from the main text and summing over all water absorption bands, and (b) total overlap integral between fluorophore emission and water absorption for all investigated fluorophores obtained by summing over all water absorption bands.

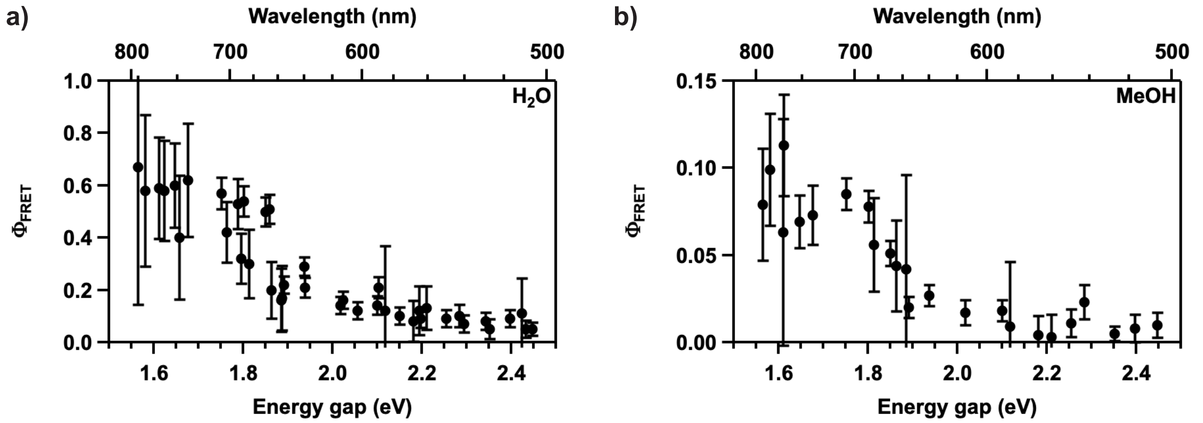

Figure S18. FRET efficiency (Φ_{FRET}) for the investigated fluorophores in (a) water and (b) methanol.

8. Supplementary References

S1 G. A. Crosby and J. N. Demas, *J. Phys. Chem.*, 1971, **75**, 991-1024.

S2 A. Fürstenberg and E. Vauthey, *Photochem. Photobiol. Sci.*, 2005, **4**, 260-267.

S3 K. Klehs, C. Spahn, U. Endesfelder, S. F. Lee, A. Fürstenberg and M. Heilemann, *ChemPhysChem*, 2014, **15**, 637-641.

S4 A. Sillen and Y. Engelborghs, *Photochem. Photobiol.*, 1998, **67**, 475-486.

S5 J. R. Lakowicz, *Principles of Fluorescence Spectroscopy*, Kluwer Academic, New York, Second edn., 1999.

S6 W. C. Waggener, *Anal. Chem.*, 1958, **30**, 1569-1570.

S7 H. Morita and S. Nagakura, *J. Mol. Spectrosc.*, 1974, **49**, 401-413.

S8 R. A. J. Litjens, T. I. Quickenden and C. G. Freeman, *Appl. Opt.*, 1999, **38**, 1216-1223.

S9 W. W. Parson, *Modern Optical Spectroscopy*, Springer, Berlin, Heidelberg, New York, 2007.

S10 S. E. Braslavsky, E. Fron, H. B. Rodriguez, E. S. Roman, G. D. Scholes, G. Schweitzer, B. Valeur and J. Wirz, *Photochem. Photobiol. Sci.*, 2008, **7**, 1444-1448.

S11 M. J. Abraham, T. Murtola, R. Schulz, S. Páll, J. C. Smith, B. Hess and E. Lindahl, *SoftwareX*, 2015, **1-2**, 19-25.

S12 M. J. Frisch, G. W. Trucks, H. B. Schlegel, G. E. Scuseria, M. A. Robb, J. R. Cheeseman, G. Scalmani, V. Barone, G. A. Petersson, H. Nakatsuji, X. Li, M. Caricato, A. V. Marenich, J. Bloino, B. G. Janesko, R. Gomperts, B. Mennucci, H. P. Hratchian, J. V. Ortiz, A. F. Izmaylov, J. L. Sonnenberg, D. Williams-Young, F. Ding, F. Lipparini, F. Egidi, J. Goings, B. Peng, A. Petrone, T. Henderson, D. Ranasinghe, V. G. Zakrzewski, J. Gao, N. Rega, G. Zheng, W. Liang, M. Hada, M. Ehara, K. Toyota, R. Fukuda, J. Hasegawa, M. Ishida, T. Nakajima, Y. Honda, O. Kitao, H. Nakai, T. Vreven, K. Throssell, J. A. Montgomery Jr., J. E. Peralta, F. Ogliaro, M. J. Bearpark, J. J. Heyd, E. N. Brothers, K. N. Kudin, V. N. Staroverov, T. A. Keith, R. Kobayashi, J. Normand, K. Raghavachari, A. P. Rendell, J. C. Burant, S. S. Iyengar, J. Tomasi, M. Cossi, J. M. Millam, M. Klene, C. Adamo, R. Cammi, J. W. Ochterski, R. L. Martin, K. Morokuma, O. Farkas, J. B. Foresman and D. J. Fox, *Journal*, 2016.

S13 J. Wang, R. M. Wolf, J. W. Caldwell, P. A. Kollman and D. A. Case, *J. Comput. Chem.*, 2004, **25**, 1157-1174.

S14 A. W. Sousa da Silva and W. F. Vranken, *BMC Res. Notes*, 2012, **5**, 367.

S15 W. L. Jorgensen, J. Chandrasekhar, J. D. Madura, R. W. Impey and M. L. Klein, *J. Chem. Phys.*, 1983, **79**, 926-935.

S16 U. Essmann, L. Perera, M. L. Berkowitz, T. Darden, H. Lee and L. G. Pedersen, *J. Chem. Phys.*, 1995, **103**, 8577-8593.

S17 B. Hess, *J. Chem. Theory Comput.*, 2008, **4**, 116-122.

S18 G. Bussi, D. Donadio and M. Parrinello, *J. Chem. Phys.*, 2007, **126**, 014101.

S19 M. Mezei and D. L. Beveridge, *Methods Enzymol.*, 1986, **127**, 21-47.

S20 W. Song, R. Biswas and M. Maroncelli, *J. Phys. Chem. A*, 2000, **104**, 6924-6939.

S21 S. F. Lee, Q. Vérolet and A. Fürstenberg, *Angew. Chem. Int. Ed.*, 2013, **52**, 8948-8951.

S22 S. Reindl, A. Penzkofer, S. H. Gong, M. Landthaler, R. M. Szeimies, C. Abels and W. Baumler, *J. Photochem. Photobiol. A*, 1997, **105**, 65-68.

S23 Y. Marcus, *Chem. Soc. Rev.*, 1993, **22**, 409-416.

S24 C. L. Braun and S. N. Smirnov, *J. Chem. Ed.*, 1993, **70**, 612.

S25 R. M. Pope and E. S. Fry, *Appl. Opt.*, 1997, **36**, 8710-8723.
